# Investigation of enhanced Am selectivity for Eu in solvent extraction using a BTPhen ligand substituted with halogen†

**DOI:** 10.1039/d2ra05515e

**Published:** 2023-01-16

**Authors:** Yuto Fukasawa, Satoru Nakashima

**Affiliations:** a Basic Chemistry Program, Graduate School of Advanced Science and Engineering, Hiroshima University 1-3-1 Kagamiyama Higashi-Hiroshima Hiroshima 739-8526 Japan snaka@hiroshima-u.ac.jp; b Natural Science Center for Basic Research and Development (N-BARD), Hiroshima University 1-4-2 Kagamiyama Higashi-Hiroshima Hiroshima 739-8526 Japan

## Abstract

The effect of bromine (Br) on the separation of Am/Eu using BTPhen was investigated using DFT calculations. The simulated results agreed well with the reported experimental result of Br substitution. It is concluded that the contribution of the d orbital becomes less important by increasing the number of Br atoms, that is, the relative contribution of the f orbital becomes important. The Am f orbital contributed to both bonding and antibonding interactions with the ligand, whereas the Eu f orbital contributed to the antibonding interaction with the ligand. To study the halogen effect systematically, we introduced a series of new halogen atoms (chlorine, fluorine, iodine) into BTPhen. When the electronegativity of the halogen atom increases, the Δ*G* for complex formation shifts to the positive direction, and the ΔΔ*G* which shows the difference in Δ*G* between Am and Eu becomes a large negative value, suggesting that the Am selectivity is larger. This is due to the increased Δ*ρ*_BCP_ (= *ρ*_BCP_(Am) − *ρ*_BCP_(Eu)) between the metal and the ligating nitrogen atom with an increase of electronegativity of the halogen atom.

## Abstract 2

When the electronegativity of the substituted halogen atom for BTPhen ligand increases, Δ*G*(Eu) shifts to a more positive direction than Δ*G*(Am), ΔΔ*G* becomes more negative and the Am selectivity increases.

## Graphical abstract

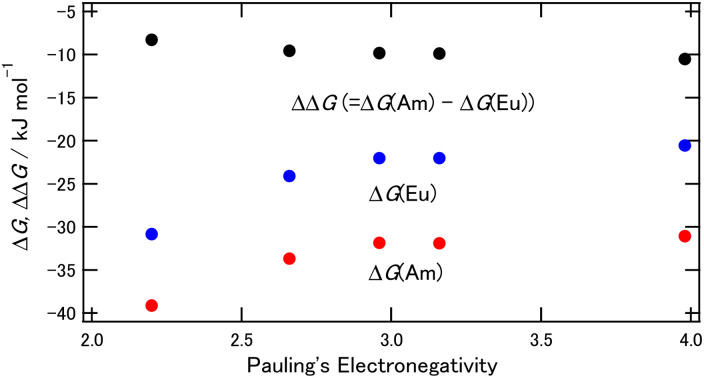

## 1. Introduction

Treatment of spent nuclear fuel (SNF), which is generated from nuclear power plants, is one of the current issues in the world. Important ingredients (*e.g.*, uranium and plutonium) in SNF can be recovered within a particular step of the nuclear fuel cycle, and the residues after this step become a high-level liquid waste (HLLW).^1^ The HLLW will be stored in the underground depository until the decrease in its radioactivity is satisfactory. The HLLW contains nuclides with a very long half-life, such as Np, Am, and Cm, so-called minor actinides (MA), which makes the storage period long.^2^ In order to decrease the storage period, the transmutation method, which converts the nuclide to one with a shorter half-life by irradiation with neutron rays, is applied.^3^ However, the lanthanides (Ln) which co-exist in HLLW are a problem because they inhibit the transmutation of the MA due to their higher abundance, and their larger reaction cross-section for neutrons than MA. The chemical character of Ln is similar to that of MA. The situation makes the selective separation of MA from Ln important.^4^ Solvent extraction methods have attracted attention as one of the most effective methods for selective separation.

To understand the mechanism of MA/Ln separation is important. We have already estimated the validity of density functional theory (DFT) to reproduce the bonding property of f-block compounds by comparing the experimental ^151^Eu and ^237^Np Mössbauer isomer shifts and scalar-relativistic zeroth-order regular approximation Hamiltonian (ZORA) DFT calculation.^5^ As a result, the performance of the reproducibility increased in the order of BP86, B3LYP, and B2PLYP functionals, and, in particular, the B2PLYP results are strongly correlated with the experimental results. Therefore, DFT has been employed as one of the most reliable methods to estimate the metal-extractants interaction for understanding the difference between MA and Ln ions.^6^ This was elucidated using DFT calculation that showed that the difference in f-orbital contribution between Am and Eu ions to the interaction with the extractants differentiates the bonding type, *i.e.*, the bonding interaction with sulfur and nitrogen donor atoms and the antibonding interaction with oxygen donor atoms for Am f-orbital, whereas for the Eu f-orbital a non-bonding interaction with sulfur and nitrogen donor atoms and a bonding interaction with oxygen donor atoms. However, both the Am and Eu d-orbital contributions were mainly bonding interactions which were independent of the donor atoms.

**Fig. 1:**
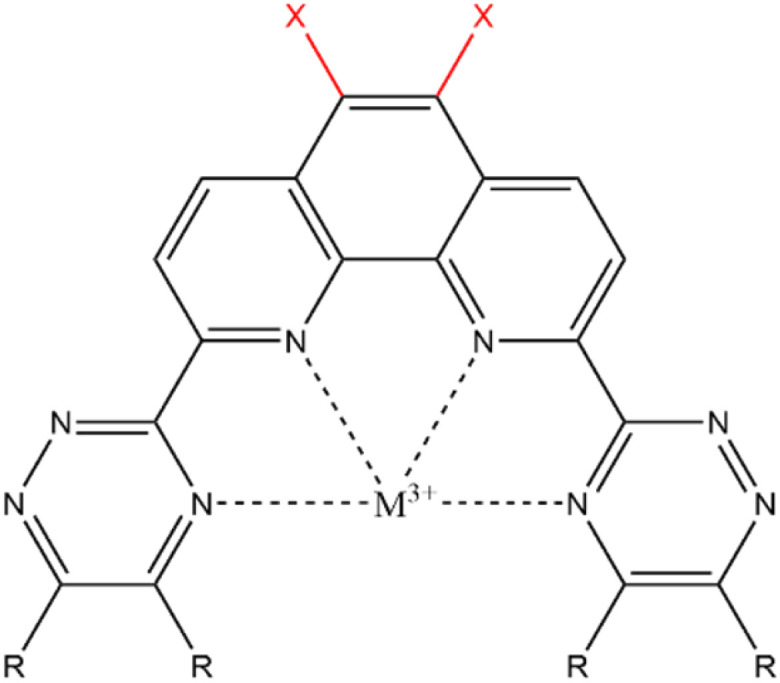
General structure of X_2_BTPhen.

Many types of ligands have been investigated for their extraction ability, and the results indicated that the ligands having nitrogen-containing aromatic ring, such as bipyridine, triazine,^7^ and phenanthroline,^8^ have shown good abilities for extraction and separation of Am. The 2,9-bis(2,4,6-triazinyl)-1,10-phenanthroline (BTPhen, Fig. 1) type ligands are the most typical ligand groups which have high selectivity.^8^ The modification of the ligand is also the strategy for the Am selectivity. Afsar *et al.* investigated the effect of the substitution of the 5- and 6-position hydrogens of BTPhen's phenanthroline ring with bromines on the Am selectivity, and they found that bromine-substituted BTPhens (HBrBTPhen, Br_2_BTPhen) have a higher separation ability than the non-substituted BTPhen.^9^ The results were caused by the lesser Eu-extraction ability of Br_2_BTPhen than the non-substituted BTPhen, but the mechanism of these changes has not yet been clarified in detail. Introduction of bromine atoms to the phenanthroline ring was expected to cause some type of modification on the electronic environment of the ligating nitrogen atoms, and to lead to the improvement of Am/Eu selectivity. The introduction of a substituent to the ligand may become a new strategy to separate Ln/MA. In the present study using DFT calculations, we tried to clarify the mechanism of change in the extraction ability of BTPhen by substituting hydrogen with bromine. Then, the substitutions by other halogens (chlorine, fluorine, and iodine) were also surveyed in order to understand the halogen effect more systematically.

## 2. Computational details

**Fig. 2:**
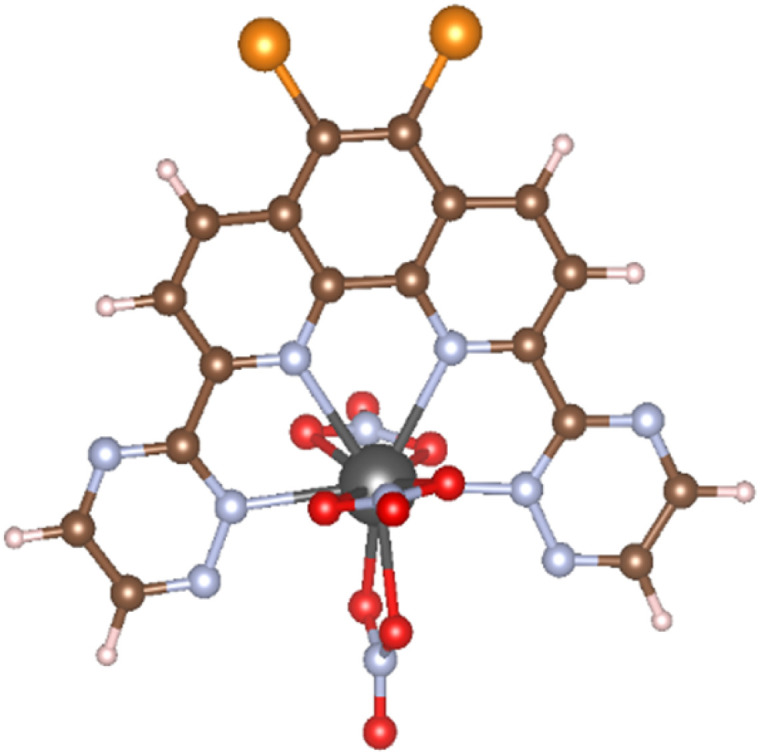
The optimised structure of [Am(Br_2_BTPhen)(NO_3_)_3_], in which black, brown, pale blue, red, orange, and pink spheres show Am, C, N, O, Br, and H atoms, respectively.

Calculation models of (X_1_,X_2_)BTPhen [(X_1_,X_2_) = (H,H), (H,Br), (Br,Br), (F,F), (Cl,Cl), (I,I)] and the complexes with Am and Eu were created by referring to the suitable single crystal X-ray structures obtained from Crystal Structure Database (CSD) software.^10^ We used [Eu(CyMe_4_-4,7-dimethyl-BTPhen)(NO_3_)_3_] (CSD reference code: ZAYHOZ)^11^ as the initial coordination model for creating calculation models, and [M((X_1_,X_2_)BTPhen)(NO_3_)_3_] (M = Am, Eu; (X_1_,X_2_) = (H,H), (H,Br), (Br,Br), (F,F), (Cl,Cl), (I,I)) (Fig. 2) were created by deleting some side chains and replacing the 5,6-position hydrogen atoms of the phenanthroline ring with halogen atoms. The reported crystal structure of [Gd(H_2_O)_9_](CF_3_SO_3_)_3_ (CSD reference code: BUVVOD)^12^ was also used to make the models of non-aqua complex, [M(H_2_O)_9_]^3+^ (M = Am, Eu).

The complex formation reaction, which is shown in eqn (1), was considered by determining the Gibbs energy difference under standard conditions (298.15 K, 1 atm).

1 [M(H_2_O)_9_]^3+^ + (X_1_,X_2_)BTPhen + 3NO_3_^−^ → 9H_2_O + [M((X_1_,X_2_)BTPhen)(NO_3_)_3_]

The Gibbs energy difference, Δ*G*(M)(X_1_,X_2_), and the difference between Am and Eu, ΔΔ*G*(X_1_,X_2_), are represented by eqn (2) and eqn (3), respectively.

2 Δ*G*(M)(X_1_,X_2_) = {9*G*(H_2_O) + *G*([M((X_1_,X_2_)BTPhen)(NO_3_)_3_])} − {*G*([M(H_2_O)_9_]^3+^)+ *G*((X_1_,X_2_)BTPhen) + 3*G*(NO_3_^−^)}

3 ΔΔ*G*(X_1_,X_2_) = Δ*G*(Am)(X_1_,X_2_) − Δ*G*(Eu)(X_1_,X_2_)

Eqn (4) to (6) were used for the calculation of the Gibbs energy. The contributions to the Gibbs energy from vibrations were calculated as a quasi-harmonic approximation, whereas low-frequency vibrational modes less than 60 cm^−1^ were increased to 60 cm^−1^.^13,14^

4 *G* = *H* − *TS*

5 *H* = *U* + *k*_B_*T* = *E*_tot_ + *E*_ZPE_ + *E*_vib_ + *E*_trs_ + *k*_B_*T*

6 *S* = *S*_spin_ + *S*_vib_ + *S*_rot_ + *S*_trs_

Bonding energy calculations were also performed. The calculation method and the results are shown in the ESI.† The *E*_BSSE_ obtained was large, and although the exact reason for this was not revealed, the values of the Am complexes were systematically larger than the values of the corresponding Eu complexes.

All the DFT calculations were executed using ORCA program package ver. 4.1.0;^15^ for further information, see our previous studies.^6^ The scalar-relativistic effect was considered using a ZORA Hamiltonian^16,17^ with segmented all-electron relativistically contracted (SARC) basis sets. The SARC basis sets were used as the contractions of {91^20^/81^12^/71^9^/61^6^} for Am^18^ and of {61^17^/51^11^/41^8^/41^2^} for Eu,^19^ for both geometry optimisation and single-point energy calculation. The geometry optimisation calculations were followed by normal frequency mode calculations, carried out by using the BP86 functional^20,21^ with assigned TZVP basis sets for the metal center, and SVP basis sets for the other atoms.^22^ The B2PLYP functional^23^ was applied for single-point energy calculations instead of BP86,^22^ whereas using the same basis sets with geometry optimisation. The solvation effect was considered using a conductor-like polarisable continuum model (C-PCM)^24^ for all the DFT calculations. All of the electronic ground states of Eu and Am were considered as a spin septet by using the spin-unrestricted Kohn–Sham procedure. All the converged structures were confirmed to be local minima by using a normal frequency mode calculation. Resolution of the identity approximation was employed for all self-consistent field (SCF) calculations.^25,26^ Details of the conditions of the SCF calculations are indicated in the ESI.† The three-dimensional descriptions of coordination geometry and molecular orbital were visualised and modified by using VESTA^27^ and Winmostar^28^ programs. Multiwfn ver. 3.8 (ref. 29) and NBO ver. 7.0 (ref. 30) were used for population analysis.

## 3. Results and discussion

### 3.1 Effect of Br substitution

It was reported that the Am selectivity increased by introducing Br atoms in the 5- and 6-positions of the BTPhen.^9^ In this section, the effect of Br substitution on the Am selectivity using DFT calculation is discussed.

#### 3.1.1. Structural analysis of the optimised structure

We calculated the interatomic distances between the metal center and the nitrogen atom bonding to the metal center, and the dihedral angle which consists of four nitrogen atoms ligated to the metal of BTPhen. Table 1 shows the results from the structural analysis. The interatomic distances were slightly decreased after the Br substitution, and in particular the magnitude of the decrease was larger for Am than for Eu as shown by the value of Δ*L*_M–N_ shown in Table 1. The difference between the dihedral angles of the donor atoms before and after complexation also indicated the same trend as that of the interatomic distances, as shown by the value of ΔΔ*ω* shown in Table 1. This trend seems to indicate that the Br substitution of BTPhen made it easier to form a complex with metals, which is more characteristic for Am.

**Table 1:** Averaged metal to donor atom interatomic distance *L*_M–N_, difference of dihedral angle of donor atoms before and after complexation Δ*ω*, and the difference between them when compared with the non-substituted H_2_BTPhen complex of the same metal-center, Δ*L*_M–N_ and ΔΔ*ω*

Complex		*L*_M–N_/Å	Δ*L*_M–N_/Å	Δ*ω*/degree	ΔΔ*ω*/degree
M = Eu	X_1_,X_2_ = H,H	2.596	—	5.265	—
	X_1_,X_2_ = H,Br	2.596	0.00	4.421	−0.844
	X_1_,X_2_ = Br,Br	2.594	−0.02	3.755	−1.510
M = Am	X_1_,X_2_ = H,H	2.595	—	5.392	—
	X_1_,X_2_ = H,Br	2.593	−0.02	3.025	−2.367
	X_1_,X_2_ = Br,Br	2.591	−0.04	2.597	−2.795

#### 3.1.2. Energy calculation of (X_1_,X_2_) BTPhen complexes [(X_1_,X_2_) = (H,H), (H,Br), (Br,Br)]

Table 2 shows the results of the Gibbs energy calculation for the complex formation. This indicates that the Δ*G*(M) values shift to the positive direction, with the increase of the bromine substitution number. This suggests that the Br_2_BTPhen forms a less stable complex with Am and Eu than H_2_BTPhen, and this seems to be contradictory to the results of the structural analysis. It is considered that the electron withdrawing Br decreased the coordination ability of BTPhen. The shortening of *L*_M–N_ might be explained by the increased back donation from the metal to the ligand. However, the ΔΔ*G*(X_1_,X_2_) values shift to the negative direction with the increase of the bromine substitution number. A negative value of ΔΔ*G*(X_1_,X_2_) means that the ligand exhibits more Am selectivity, and these results suggest that the increase of the bromine substitution number caused the decrease of the stability of the complexes, whereas the Am/Eu selectivity was enhanced by a larger decrease in stability of the Eu complex than that of the Am. The results from the energy calculation explain the experimental results,^9^ where Br_2_BTPhen has less Eu-extraction ability than the non-substituted BTPhen.

**Table 2:** Complex formation energy Δ*G*(M)(X_1_,X_2_) and its difference ΔΔ*G*(X_1_,X_2_)

Ligand	Δ*G*(M)(X_1_,X_2_)/kJ mol^−1^		ΔΔ*G*(X_1_,X_2_)/kJ mol^−1^
	M = Eu	M = Am	
X_1_,X_2_ = H,H	−30.84	−39.12	−8.28
X_1_,X_2_ = H,Br	−25.18	−34.40	−9.22
X_1_,X_2_ = Br,Br	−22.02	−31.84	−9.83

The binding energies of the BTPhen complexes, shown in Table S5 (ESI) and Fig. S1 (ESI),† are increased by changing H into Br for the Am complexes, whereas the binding energies did not significantly change for the Eu complexes, although the binding energies for the Eu complexes are slightly larger than that for Am. The results suggest that the binding energy of [M(H_2_O)_9_]^3+^ is different between Eu and Am which explains why the Δ*G* values and the binding energy for the Am complex shift to the negative direction by introducing Br, and suggest increased Am selectivity.

#### 3.1.3. Bond analysis of (X_1_,X_2_)BTPhen complexes [(X_1_,X_2_) = (H,H), (H,Br), (Br,Br)]

Table 3 shows the electron density at the bond critical point (*ρ*_BCP_) of each BTPhen complex. The comparison of the *ρ*_BCP_ values for Table 3 suggests the degree of the relative bond strength. The bond having a larger *ρ*_BCP_ value would be recognised as a stronger bond. There are differences found between Eu and Am when using the same ligand. This trend clearly indicates the well-known ability of ligands to create stronger interactions with Am than with Eu. Then, there was a slight change by introducing Br for each ligand for the same metal center, *i.e.*, the Δ*ρ*_BCP_ (= *ρ*_BCP_(Am) – *ρ*_BCP_(Eu)) slightly increases by introducing Br. These results suggest that increasing the bromine substitution number slightly affects the strength of those bonds. It was shown that the Am selectivity is increased by increasing the number of Br atoms. Table 4 shows the natural electron population *ρ*_atom_(M), natural atomic spin population *ρ*_spin_(M), and the electron configuration of Am and Eu for each complex, which were the results from the natural population analysis.^31^

**Table 3:** Electron density at bond critical point *ρ*_BCP_ of [M(X_1_X_2_BTPhen)(NO_3_)_3_]

Complex	*ρ*_BCP_	
	Eu	Am
X_1_,X_2_ = H,H	0.0389	0.0442
X_1_,X_2_ = H,Br	0.0389	0.0444
X_1_,X_2_ = Br,Br	0.0391	0.0446

**Table 4:** Natural population analysis of [M(X_1_X_2_BTPhen)(NO_3_)_3_]

Complex		*ρ*_atom_ (M)	*ρ*_spin_ (M)	Electron configuration
M = Eu	X_1_,X_2_ = H,H	1.168	5.885	[_54_Xe] 6s(0.23) 4f(6.17) 5d(0.97) 6p(0.01)
	X_1_,X_2_ = H,Br	1.165	5.886	[_54_Xe] 6s(0.23) 4f(6.17) 5d(0.97) 6p(0.01)
	X_1_,X_2_ = Br,Br	1.159	5.887	[_54_Xe] 6s(0.23) 4f(6.17) 5d(0.98) 6p(0.01)
M = Am	X_1_,X_2_ = H,H	1.182	5.814	[_86_Rn] 7s(0.24) 5f(6.32) 6d(0.98) 7p(0.01)
	X_1_,X_2_ = H,Br	1.195	5.812	[_86_Rn] 7s(0.24) 5f(6.32) 6d(0.97) 7p(0.01)
	X_1_,X_2_ = Br,Br	1.191	5.811	[_86_Rn] 7s(0.24) 5f(6.32) 6d(0.97) 7p(0.01)

The larger *ρ*_atom_(M) values for Am than for Eu reveal the Am selectivity. The *ρ*_atom_(Eu) values decrease with the increasing bromine substitution number, whereas the *ρ*_spin_(Eu) increased. The *ρ*_atom_(Am) values either increased or there was relatively little or no change with the increasing Br substitution number, whereas the *ρ*_spin_ (Am) decreased. The tendency of *ρ*_spin_(M) was systematically reversed to that of *ρ*_atom_(M). The dependence on Br substitution is opposite between Am and Eu. That is, the back donation is increased by the introduction of Br for Am, whereas it is decreased by the introduction of Br for Eu. These results are in agreement with the results from the energy calculation. Then, the analyzed electron configuration also exhibited the Am selectivity of BTPhens, because the 5f orbitals of Am received more electrons than the 4f orbitals of Eu. However, there was no significant change in electron configuration when changing the number of Br atoms for both Eu and Am.

**Fig. 3:**
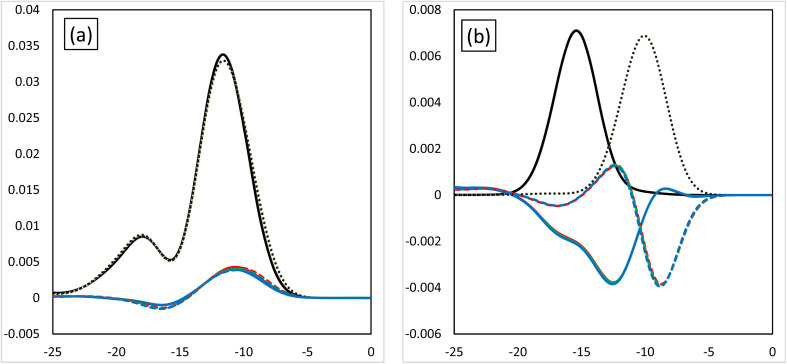
The MOOP diagrams of each orbital. (a) The d-orbital and (b) the f-orbital. Black solid line: the PDOS of the Eu atoms of each orbital. Black dotted line: the PDOS of the Am atoms of each orbital. The coloured lines are the OPDOS of the Am or Eu atoms of each orbital. Red: (X_1_,X_2_) = (H,H), green: (X_1_,X_2_) = (H,Br), blue: (X_1_,X_2_) = (Br,Br).

Fig. 3 shows the MOOP diagrams obtained from density of states (DOS) analysis using Mulliken's method.^32^Fig. 3(a) and (b) show the results for the d- and f-orbitals, respectively. The black lines indicate the partial DOS (PDOS) of each metal. The coloured lines indicate the overlap population DOS (OPDOS) between the metal d- or f-orbitals and all the orbitals of the N-donor atoms in the valence α-spin orbital region. The OPDOS from the H_2_BTPhen, BrHBTPhen, and Br_2_BTPhen complexes are indicated as red, green, and blue lines, respectively. The solid and dotted lines represent PDOS or OPDOS related to the Eu and Am complexes, respectively. For the d-orbital (Fig. 3(a)), a notable PDOS distribution appeared at −5 to −20 eV energy region, and the OPDOS distribution also appeared at the same region and was mainly positive. There was almost no difference among each combination of metals and ligands, thus this feature of d-orbitals was suggested to have largest contribution to form each complex by causing metal-donor bonding type interactions, when compared with the f-orbitals. When we compare (X_1_,X_2_) = (H,H), (H,Br), and (Br,Br), it is shown that this bonding characteristic slightly decreases with the increasing number of Br atoms. Fig. 3(b) shows the results from the analysis relating to f-orbitals, which show DOS curves with different trends depending on the metals used. For the Eu complexes, PDOS distributions were observed at the −10 to −20 eV energy region, whereas OPDOS distributions were also seen in the region to have only a negative sign. On the other hand, for the Am, the PDOS distributions appeared at the −5 to −15 eV energy region, whereas the OPDOS distribution was also shown at the region with negative and positive signs at the same time. The selectivity of Am/Eu is determined by the f-orbital features. For Eu, this shows an antibonding character, whereas for Am it shows both bonding and antibonding characteristics. The difference shows Am selectivity, which is in accordance with our previous results.^6^ By considering all OPDOS, it is concluded that the contribution of the d-orbital becomes less important when increasing the number of Br atoms, therefore, the relative contribution of the f-orbital becomes more important for the separation of Am.

### 3.2 Effect of halogen substitution

The effect of Br introduction was shown in the last section, but the effect is slight. To confirm the effect of halogen substitution, a systematic study of the halogen effect is performed in the present section.

#### 3.2.1. Structural analysis of the optimised structure

Table 5 is a summary of the structural analysis comparing the effects of substitution for a series of halogens. When the X = F, the *L*_M–N_ values become longer for both Am and Eu complexes than for the non-substituted complexes. The change for Eu is slightly larger than that for Am with fluorine. The shortest value of *L*_M–N_ is observed for X = Br for both of the Am and Eu complexes. Then, the Δ*ω* values show a decrease for all the halogen-substituted complexes compared to the non-substituted complexes. These Δ*L*_M–N_ and ΔΔ*ω* changes tend to be slightly larger for Am complexes with Br, Cl, and I.

**Table 5:** Averaged metal to donor atom interatomic distance *L*_M–N_, difference of dihedral angle of donor-atoms before and after complexation Δ*ω*, and their difference between them when compared with the non-substituted H_2_BTPhen complex of the same metal-center, Δ*L*_M–N_ and ΔΔ*ω*

Complex		*L*_M–N_/Å	Δ*L*_M–N_/Å	Δ*ω*/degree	ΔΔ*ω*/degree
M = Eu	X = H	2.596	—	5.265	—
	X = F	2.602	0.006	4.461	−0.804
	X = Cl	2.596	0.000	3.921	−1.345
	X = Br	2.594	−0.002	3.755	−1.510
	X = I	2.595	−0.001	4.350	−0.915
M = Am	X = H	2.595	—	5.392	—
	X = F	2.596	0.001	3.285	−2.107
	X = Cl	2.592	−0.003	2.733	−2.659
	X = Br	2.591	−0.004	2.597	−2.795
	X = I	2.593	−0.002	3.191	−2.201

#### 3.2.2. Energy calculations of the X_2_BTPhen complexes [X = Br, Cl, F, H, I]

**Fig. 4:**
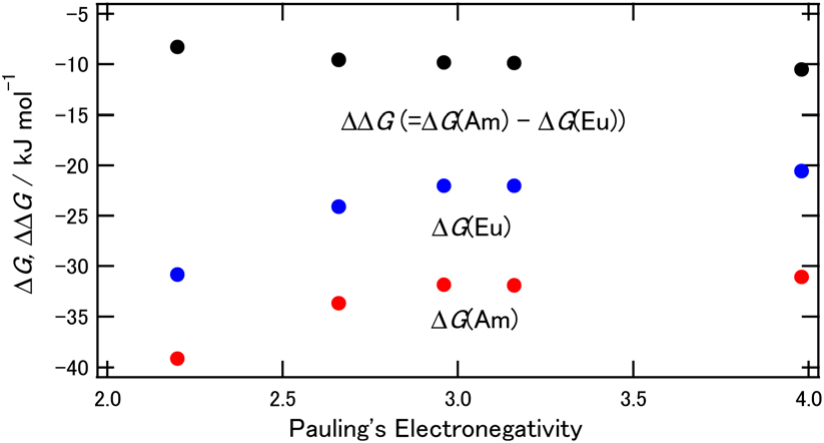
The Δ*G* and ΔΔ*G* dependence on the halogens of the 5- and 6-positions of BTPhen. Red: Δ*G* of M = Am, blue: Δ*G* of M = Eu, black: ΔΔ*G*.

A comparison of the results from the energy calculations for a series of halogen substitutions are shown in Fig. 4, and the corresponding numerical data are provided in Table S2 (ESI†). The Δ*G*(M) values shift to the positive direction together with the increasing electronegativity of the substituted halogens, and this means that the complexes become less stable when the electronegative halogen is introduced. On the other hand, the ΔΔ*G* values showed an inverse trend of Δ*G*(M), which is the same for the results shown in Tables 2, *i.e.*, ΔΔ*G* (= Δ*G*(Am) − Δ*G*(Eu)) becomes more negative in the complexes substituted by smaller halogen. Fig. 4 suggests that the halogen substitution of the BTPhens causes an increase of Am separation ability while this decreases the stability of each complex, and these phenomena are more typical for the smallest, most electronegative halogen, fluorine.

The binding energy of the BTPhen complexes, shown in Table S5 (ESI) and Fig. S2 (ESI),† increased with the Pauling's electronegativity of the halogens on the 5- and 6- positions of the BTPhen for the Am complexes, whereas the binding energy did not significantly change for the Eu complexes. The results suggest that the binding energy of [M(H_2_O)_9_]^3+^ is different between Eu and Am and this explains that the Δ*G* values and the binding energy for the Am complex shift to the negative direction by introducing a more electronegative halogen, and this is suggested as the reason for the increased Am selectivity.

#### 3.2.3. Bond analysis of X_2_BTPhen complexes [X = Br, Cl, F, H, I]

**Fig. 5:**
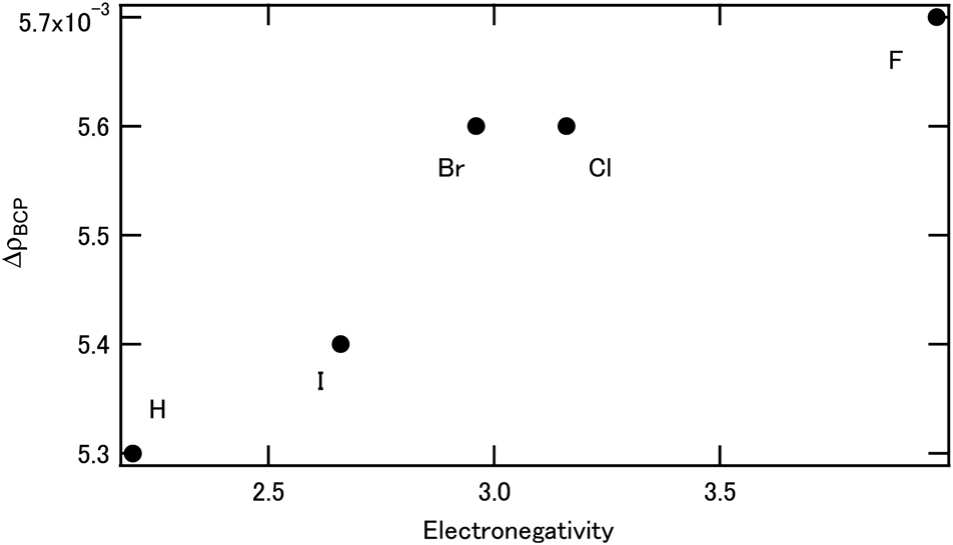
The halogen effect on the Δ*ρ*_BCP_.

Table S3† (ESI) shows electron density at the bond critical point *ρ*_BCP_ of [M(X_2_BTPhen)(NO_3_)_3_] (X = Br, Cl, F, H, and I). For the Eu, the *ρ*_BCP_ value decreased with the F substitution when compared with the non-substituted case, whereas the *ρ*_BCP_ value did not change for the other halogens. However, for Am, the *ρ*_BCP_ value did not change significantly with the F substitution compared with the non-substituted case, while the *ρ*_BCP_ value increased for the other halogens. We considered the Δ*ρ*_BCP_, which is calculated by *ρ*_BCP_(Am) − *ρ*_BCP_(Eu). Fig. 5 shows the change of Δ*ρ*_BCP_ which is dependent on Pauling's electronegativity. As can be seen in Fig. 5, the Δ*ρ*_BCP_ values increase with increasing electronegativity, which reveals the increase of Am selectivity.

Table 6 shows the results from the natural population analysis used to compare the effect of different halogens. Fluorine substitution is unique, *i.e.*, *ρ*_atom_(M) for both Eu and Am is high. Except for F substitution, the introduction of a halogen decreases the *ρ*_atom_(Eu), whereas it increases the *ρ*_atom_(Am). The trend is the same as that shown in Table 4.

**Table 6:** Natural population analysis of [M(X_2_BTPhen)(NO_3_)_3_]

Complex		*ρ*_atom_ (M)	*ρ*_spin_ (M)	Electron configuration
M = Eu	X = H	1.168	5.885	[_54_Xe] 6s(0.23) 4f(6.17) 5d(0.97) 6p(0.01)
	X = F	1.171	5.885	[_54_Xe] 6s(0.23) 4f(6.17) 5d(0.97) 6p(0.01)
	X = Cl	1.163	5.886	[_54_Xe] 6s(0.23) 4f(6.17) 5d(0.97) 6p(0.01)
	X = Br	1.159	5.887	[_54_Xe] 6s(0.23) 4f(6.17) 5d(0.98) 6p(0.01)
	X = I	1.162	5.886	[_54_Xe] 6s(0.23) 4f(6.17) 5d(0.98) 6p(0.01)
M = Am	X = H	1.182	5.814	[_86_Rn] 7s(0.24) 5f(6.32) 6d(0.98) 7p(0.01)
	X = F	1.198	5.811	[_86_Rn] 7s(0.24) 5f(6.32) 6d(0.97) 7p(0.01)
	X = Cl	1.193	5.811	[_86_Rn] 7s(0.24) 5f(6.32) 6d(0.97) 7p(0.01)
	X = Br	1.191	5.811	[_86_Rn] 7s(0.24) 5f(6.32) 6d(0.97) 7p(0.01)
	X = I	1.195	5.811	[_86_Rn] 7s(0.24) 5f(6.32) 6d(0.97) 7p(0.01)

**Fig. 6:**
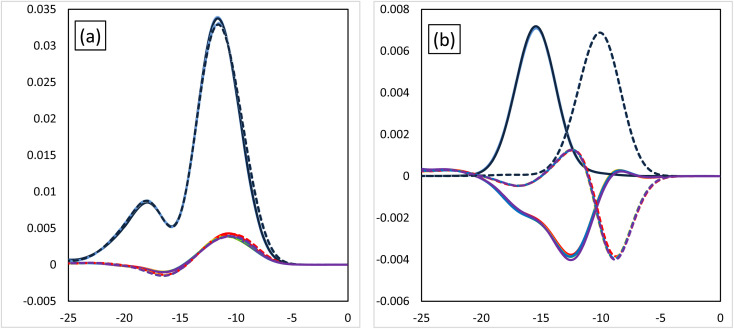
The MOOP diagrams of each orbital, (a) d-orbital and (b) f-orbital. Black solid line: PDOS of each of the Eu's orbitals, black dotted line: PDOS each of the Am's orbitals. Coloured lines: OPDOS of Am or Eu's of each orbital, red: X = H, yellow: X = F, green: X = Cl, blue: X = Br, purple: X = I.

Fig. 6 shows the MOOP diagrams, and Fig. 6(a) and (b) indicate the results of the d- and f-orbitals, respectively. The features of the contribution to the bonds (bonding/anti-bonding) are the same as those shown in Fig. 3. The diagram of d-orbitals shows a slight difference among halogens, the same as Fig. 3(a). That is, the bonding interaction decreases slightly when the halogen is introduced. However, the diagrams of the f-orbitals show a slight increase of anti-bonding contribution when the halogen is introduced for Eu, whereas the OPDOS for Am does not significantly change. These results suggest that the contribution of the d-orbital becomes less important after introducing the halogen atom, that is, the relative contribution of the f-orbital becomes more important.

## 4. Conclusions

DFT calculations were performed to reveal the halogen effect of the BTPhen type ligands on the Am selectivity. The BTPhen without side-chains was selected as the model ligand, and americium or europium were used for the metal center as representatives of the minor actinides and lanthanides, respectively. Hydrogens at the 5- and/or 6-positions of BTPhen's phenanthroline ring were substituted by bromine, in the same way as the ligands used in the reported experimental work. The result agreed well with the reported experimental results for the bromine-substituted BTPhen. It was concluded that the contribution of the d-orbital becomes less important when the number of Br atom increases, that is, the relative contribution of the f-orbital becomes more important. To investigate the effect of the halogens systematically, we introduced new halogen atoms, *i.e.*, not only bromine but also chlorine, fluorine, and iodine to the 5- and 6-positions of the phenanthroline ring of the BTPhen. When the electronegativity of the halogen atoms increases, the Δ*G* shifts to the positive direction and ΔΔ*G* becomes a large negative value, which suggests the larger selectivity of Am. This is due to the increased Δ*ρ*_BCP_ between the metal and ligating nitrogen atom for Am complexes. It is considered that the electron withdrawing halogen decreased the coordination ability of BTPhen, but the back donation from metal to ligand increased. For a series of halogen substitutions, it was also revealed that the contribution of the d-orbital becomes less important by increasing the electronegativity of the halogen atoms, and the relative contribution of the f-electron becomes more important. It was also shown that the electronic modification of the ligand by substitution contributes to the Am/Eu selectivity.

## Author contributions

Yuto Fukasawa: conceptualisation, data curation, formal analysis, funding acquisition, investigation, methodology, project administration, validation, visualisation, and writing – original draft. Satoru Nakashima: funding acquisition, investigation, project administration, resources, supervision, validation, visualisation, and writing – review and editing.

## Conflicts of interest

There are no conflicts to declare.

## Supplementary Material

RA-013-D2RA05515E-s001
