# Supplementary material for: Investigation of enhanced Am selectivity for Eu in solvent extraction using a BTPhen ligand substituted with halogen
Source: RSC Adv. 2023 Jan 16;13(4):2476–82. doi: 10.1039/d2ra05515e (PMC9841579; doi:10.1039/d2ra05515e)
Supplement: RA-013-D2RA05515E-s001 [file RA-013-D2RA05515E-s001.pdf]

Electronic Supplementary Material (ESI) for RSC Advances.
This journal is © The Royal Society of Chemistry 2022

Supplementary Information

Investigation of enhanced Am selectivity toward Eu in the solvent extraction using BTPhen ligand substituted with halogen

Yuto Fukasawa[a] and Satoru Nakashima*[a, b]

[a] *Basic Chemistry Program, Graduate School of Advanced Science and Engineering, Hiroshima University, 1-3-1, Kagamiyama, Higashi-Hiroshima, Hiroshima 739-8526, Japan.*

[b] *Natural Science Center for Basic Research and Development (N-BARD), Hiroshima University, 1-4-2, Kagamiyama, Higashi-Hiroshima, Hiroshima 739-8526, Japan*

Calculation of thermal correction to Gibbs free energy

Standard Gibbs energy (G) can be explained as shown in eq. S1, i.e. the sum of total energy (E_{tot}) and thermal correction to the Gibbs energy term ($G_{\mathrm{corr}}(T)$). The $G_{\mathrm{corr}}(T)$ can be divided into thermal correction to enthalpy term ($H_{\mathrm{corr}}(T)$) and entropy term ($S(T)$) (eq. S2). The $H_{\mathrm{corr}}(T)$ also can be divided into zero-point energy (E_{ZPE}), the contributions of vibration ($E_{\mathrm{vib}}(T)$), rotation ($E_{\mathrm{rot}}(T)$), translation ($\mathrm{E}_{\mathrm{trans}}(T)$), and Boltzmann thermal distribution ($k_{\mathrm{B}}T$, k_{B} denotes Boltzmann constant), as shown in eq. S3. The $S(T)$ also can be divided into the contributions of electron ($\mathrm{S}_{\mathrm{ele}}$), vibration ($\mathrm{S}_{\mathrm{vib}}(\mathrm{T})$), rotation, ($S_{\mathrm{rot}}(T)$) and translation ($S_{\mathrm{trans}}(T)$), as shown in eq. S4. The contributions of vibration and rotation to enthalpy and entropy are formulated based on harmonic oscillator and rigid rotator approximations, respectively. Quasi-harmonic approximation, which was the well-known breakdown of the harmonic oscillator model for Gibbs energies of low-frequency vibrational modes, was introduced in analyzing the vibrational enthalpy and entropy terms by raising the vibrational frequencies, which are less than 60 cm^{-1}. The derivation of formulas was referred to "Thermochemistry in Gaussian" by Ochterski [RS1].

$$G = E_{\mathrm{tot}} + G_{\mathrm{corr}}(T) \quad \text{(S1)}$$

$$G_{\mathrm{corr}}(T) = H_{\mathrm{corr}}(T) - TS(T) \quad \text{(S2)}$$

$$H_{\mathrm{corr}}(T) = E_{\mathrm{ZPE}} + E_{\mathrm{vib}}(T) + E_{\mathrm{rot}}(T) + E_{\mathrm{trans}}(T) + k_{\mathrm{B}}T \quad \text{(S3)}$$

$$S(T) = S_{\mathrm{ele}} + S_{\mathrm{vib}}(T) + S_{\mathrm{rot}}(T) + S_{\mathrm{trans}}(T) \quad \text{(S4)}$$

The symbols, E_{ZPE}, $E_{vib}(T)$, and $S_{vib}(T)$ in eqs. S3 and S4 are described in eqs. S5–S7, where $\Theta_v(i) = hv_i/k_B$ (h and v_i mean Planck constant and frequency of ith normal vibrational mode, respectively) denotes characteristic vibrational temperature of ith normal vibrational frequency. The $E_{rot}(T)$ of nonlinear molecules and $E_{trans}(T)$ equal to $(3/2)k_BT$. The S_{ele} is considered to correspond to spin entropy of electrons generated from spin multiplet, $2s + 1$ (s denotes spin quantum number), and equals to $k_B\{\ln(2s + 1)\}$. The $S_{rot}(T)$ is described in eq. S8, where $\Theta_r(t)$ and σ_r denote characteristic rotational temperature of $t = x, y, z$ rotational axes and rotational symmetry number, respectively. The $S_{trans}(T)$ is described in eq. S9, where m and P denote molecular weight and pressure, respectively.

$$E_{ZPE} = k_B\Sigma_i(\Theta_v(i)/2) \quad \text{(S5)}$$

$$E_{vib}(T) = k_B\Sigma_i\Theta_v(i)\{\exp(\Theta_{v,i}/T) - 1\}^{-1} \quad \text{(S6)}$$

$$S_{vib}(T) = k_B\Sigma_i[(\Theta_v(i)/T)\{\exp(\Theta_v(i) - 1)\}^{-1} - \ln\{1 - \exp(-\Theta_v(i)/T)\}] \quad \text{(S7)}$$

$$S_{rot}(T) = k_B[\ln(\pi^{1/2}/\sigma_r)\{T^{3/2}(\Theta_r(x)\Theta_r(y)\Theta_r(z))^{-1/2}\} + 3/2] \quad \text{(S8)}$$

$$S_{translation}(T) = k_B\{\ln(2\pi mk_BT/h^2)^{3/2}(k_BT/P) + 5/2\} \quad \text{(S9)}$$

Calculation details of SCF calculation

Segmented all-electron relativistically contracted basis sets (SARC basis sets) for Eu and Am were assigned as $\{61^{17}/51^{11}/41^{8}/41^{2}\}$ and $\{91^{20}/81^{12}/79^{9}/61^{6}\}$, respectively. Self-consistent field (SCF) calculation was operated under the conductor-like polarizable continuum model (C-PCM). Angular grid points in the SCF calculations are set to Lebedev 194/no final grid for geometry optimization and Lebedev 302/434 as iteration/final grid for single point calculation. Integral accuracy parameters are set to 4.34 for geometry optimization and 4.67/5.61 for single-point calculation. Special grid was additionally constructed and applied for Eu and Am with an integral accuracy of 14.0 for improving the SCF energy precisions.

Density of states analysis

We explain the analytical method of density of states (DOS) for partial DOS (PDOS) of all orbitals of Am or Eu atom and overlap population DOS (OPDOS) between Am or Eu d- and f-orbital, whose values were employed in Figure 3 and Figure 6. Mulliken population analysis method was applied for the analysis. The DOS values of the ith MO ($N(i)$) is calculated by eq. S10, where $P_{\mu\nu}$ and $S_{\mu\nu}$ denote the density matrix and the overlap matrix between basis functions ψ_μ and ψ_ν, respectively.

$$N(i) = \Sigma_\mu \Sigma_\nu \mathrm{P}_{\mu\nu}(i) S_{\mu\nu} \quad \text{(S10)}$$

PDOS of metal *d*- or *f*-orbital corresponds to values when the both ψ_μ and ψ_ν functions belong to metal *d*- or *f*-orbitals. OPDOS corresponds to values when the ψ_μ and ψ_ν functions belong to metal *d*- or *f*-orbitals and atomic orbitals of the donor atoms of the ligands, respectively.

Calculation of binding energy (BE) of metal centers

The BE (E_{BE}) is defined in eq. S11 [RS2][RS3].

$$E_{\mathrm{BE}} = E_{\mathrm{comp}} - E_{\mathrm{metal}} - E_{\mathrm{ligands}} + E_{\mathrm{BSSE}} \quad \text{(S11)}$$

BSSE energy (E_{BSSE}) is calculated using eq. S12.

$$E_{\mathrm{BSSE}} = (E_{\mathrm{metal}} + E_{\mathrm{ligands}}) - (E'_{\mathrm{metal}} + E'_{\mathrm{ligands}}) \quad \text{(S12)}$$

E'_{metal} and E'_{ligands} mean single point electronic energy of metals with basis sets (so-called ghost orbitals) of ligands and that of ligands with basis sets of metal, respectively. Coordinations of metals and ligands are prepared by just removing ligands and metals, respectively, and any geometrical optimization is not operated. The results of calculations are shown in Table S5. The trend is shown in Figures S1 and S2.

References

RS1) J. W. Ochterski, *Thermochemistry in Gaussian, Gaussian Inc Pittsburgh PA.* 2000, **264**, 1–19.

RS2) M. S. Islam, R. A. Pethrick, D. Pugh and M. J. Wilson, *J. Chem. Soc., Faraday Trans.*, 1997, **93**, 387-392.

RS3) F. B. van Duijneveldt,' J. G. C. M. van Duijneveldt-van de Rijdt, and J. H. van Lenthe, *Chem. Rev.* 1994, **94**, 1873-1885.

Table S1 Numerical data of thermodynamic parameters (hartree)

Compounds	E_{tot}	U_{corr}	H_{corr}	$T*S$	G_{corr}
$Eu(H_2O)_9$	-11765.921	152.757	153.350	41.703	111.647
$Am(H_2O)_9$	-32770.995	152.762	153.355	42.424	110.930
$Eu(H_2BTPhen)(NO_3)_3$	-13049.524	209.901	210.494	52.810	157.684
$Am(H_2BTPhen)(NO_3)_3$	-34054.602	210.156	210.749	52.898	157.851
$Eu(BrHBTPhen)(NO_3)_3$	-15668.898	204.849	205.441	55.322	150.119
$Am(BrHBTPhen)(NO_3)_3$	-36673.976	204.778	205.371	55.274	150.097
$Eu(F_2BTPhen)(NO_3)_3$	-13248.229	201.159	201.751	55.431	146.320
$Am(F_2BTPhen)(NO_3)_3$	-34253.307	201.237	201.830	55.630	146.199
$Eu(Cl_2BTPhen)(NO_3)_3$	-13972.863	199.876	200.468	56.460	144.009
$Am(Cl_2BTPhen)(NO_3)_3$	-34977.941	199.833	200.426	56.709	143.717
$Eu(Br_2BTPhen)(NO_3)_3$	-18288.272	199.225	199.818	57.681	142.136
$Am(Br_2BTPhen)(NO_3)_3$	-39293.350	199.180	199.773	57.980	141.793
$Eu(I_2BTPhen)(NO_3)_3$	-27409.499	200.944	201.536	57.501	144.036
$Am(I_2BTPhen)(NO_3)_3$	-48414.577	200.886	201.479	57.767	143.712
$H_2BTPhen$	-1130.017	169.820	170.413	32.586	137.826
BrHBTPhen	-3749.393	164.493	165.086	34.970	130.115
$F_2BTPhen$	-1328.725	160.977	161.569	35.026	126.543
$Cl_2BTPhen$	-2053.359	159.650	160.242	36.086	124.156
$Br_2BTPhen$	-6368.768	158.971	159.564	37.410	122.153
$I_2BTPhen$	-15489.994	160.679	161.271	37.243	124.028
H_2O	-76.481	14.654	15.247	11.475	3.772
NO_3^-	-280.663	10.647	11.240	12.842	-1.603

Table S2 Complex formation energy ΔG(M)(X) and its difference $\Delta\Delta G$(X)

Ligand	ΔG(M)(X) / kJ mol^{-1}		$\Delta\Delta G$(X) / kJ mol^{-1}
	M= Eu	M = Am	
X = H	-30.84	-39.12	-8.28
X = F	-20.55	-31.08	-10.53
X = Cl	-22.01	-31.89	-9.88
X = Br	-22.01	-31.84	-9.83
X = I	-24.10	-33.68	-9.58

Table S3 Electron density at bond critical point ρ_{BCP} of $[M(X_2BTPhen)(NO_3)_3]$

Complex	ρ_{BCP}	
	Eu	Am
X = H	0.0389	0.0442
X = F	0.0384	0.0441
X = Cl	0.0389	0.0445
X = Br	0.0390	0.0446
X = I	0.0390	0.0444

Table S4 Coordinates of the optimized structures

36

Coordinates from ORCA-job (Br2BTPhen)_num_water_BP_ORCA4

N	8.92368738926556	4.54726994893091	3.18482080361022
N	9.03814350125903	2.04654816650362	7.11141406281773
N	10.71607212834653	2.67700178144475	2.26973897807779
N	8.01112973377108	4.19543946339495	5.74255890872676
N	9.54942309342966	1.00057994219146	7.76672834689396
N	11.59306331341334	1.76493854312355	1.84081370831586
N	7.70229892739656	2.65191258171566	9.01712653745249
N	10.86900127171783	3.87446884740702	0.19056935408529
C	6.59963297793693	7.46937204185668	3.48057918923499
C	6.13414608506936	7.28900952937600	4.77420361864298
C	6.15674771196971	5.95047720571679	6.91583402744417
C	8.21870828028560	1.60707433456857	9.66378922546260
C	9.15746601127026	0.76827403583521	9.02981594280002
C	7.58791446979663	4.00361568501429	6.99549684491061
C	8.99275091241823	5.77217580312483	1.09961661345312
C	10.38186897253412	3.69370120752526	1.44202408281558
C	8.07370363817398	6.69213969220893	1.58265230986612
C	12.11204195525007	1.88831896685660	0.60868886313458
C	8.14021156378411	2.83356146794808	7.74778986036120
C	9.39456253388757	4.70045122871787	1.94356770969373
C	6.59251537303228	6.18130579117794	5.58281389734865
C	11.74435782354391	2.96093284515385	-0.22860648823055
C	7.55720335763261	6.55441900368854	2.89958996566984
C	6.64987385480391	4.86618601991662	7.62675410861413
C	7.54248666193031	5.24614989957321	5.03926848448137
C	8.03326425412811	5.43603153428524	3.67011417740014
H	9.40572755893769	5.85983645365816	0.08621839638667
H	6.33124914098866	4.66688729937147	8.65822225250322
H	7.73923423681089	7.52842760951347	0.95296023440991
H	5.43192587405593	6.63563150782618	7.37738679965107
H	12.83044052818360	1.11559897071109	0.29139582160713
Br	4.87444111205761	8.49749986391866	5.53002914717783
Br	5.99156048224049	8.93078462412333	2.42571206401120

H	9.60183091491229	-0.10282118648790	9.53688610986101
H	12.16680586016792	3.07028900130837	-1.24270989195310
H	7.89050249559757	1.42140528880072	10.70144893326166

36

Coordinates from ORCA-job (BrHBTPhen)_V3_num_water_BP_ORCA4

N	8.93542740737865	4.57872213615781	3.18688533144807
N	9.00425655706829	2.04885208608257	7.14231742883642
N	10.78855181808869	2.76656839785125	2.28051978975502
N	8.01108529468315	4.20014243094906	5.75704762443240
N	9.50177071592235	1.00089966971001	7.80494357289949
N	11.67079115036584	1.86228689961107	1.84554555345819
N	7.65334907516881	2.66659955807616	9.03285569600597
N	10.82847558357257	3.89986354708842	0.16061462600085
C	6.58804413356541	7.46845972090293	3.51026583775956
C	6.12135919460567	7.28391960809614	4.79186044194139
C	6.12896254902937	5.95813400274394	6.90827017139666
C	8.15532464397216	1.61901676062332	9.68642244772871
C	9.09495222340108	0.77272739817067	9.06386667155672
C	7.56589040970935	4.01428747146944	7.00509002143147
C	8.98377065150133	5.81181633849271	1.10274810474626
C	10.39411126572622	3.74610605159303	1.43489689467907
C	8.06052579899325	6.72400377980196	1.59360054968703
C	12.13461459911752	1.95675627192292	0.58933077227053
C	8.10479794569823	2.84412783610804	7.76713863046410
C	9.39867350020517	4.74210869120126	1.94391667175253
C	6.58775723605578	6.17929783352679	5.58116250982299
C	11.70855786606034	2.99367996369841	-0.26508291333965
C	7.55076657639260	6.57530101689659	2.91012675444458
C	6.61539511993098	4.87529037741585	7.62650928291579
C	7.54509712363941	5.25073370675639	5.04884735429042
C	8.03620276396762	5.45574289469253	3.68045589210846
H	9.39507087755967	5.90075117158487	0.08873994834591
H	6.28737899429835	4.67001070775891	8.65406246935880
H	7.71849724273178	7.56384807894936	0.97154071749299
H	5.39396811472412	6.64648223338446	7.35268422976770

H	12.85853525624004	1.19125190861607	0.26752301035847
H	5.38731278249048	7.97678824261385	5.22826186872465
Br	5.92828407809499	8.95736142633987	2.50922414805351
H	9.52863723738684	-0.10065961534840	9.57637380024874
H	12.08912904648666	3.08151338851964	-1.29766460472096
H	7.81409416616723	1.43773900794209	10.72062569387718

36

Coordinates from ORCA-job (BTPhen)_V5_num_water_BP_ORCA4

N	8.93873344337812	4.56456347164588	3.17607822004555
N	9.01410736341867	2.07125778281590	7.16657196679366
N	10.78009361755813	2.75519257377971	2.24316367812102
N	8.01338034885264	4.20674606569015	5.76092134537567
N	9.50817942516890	1.02635391634013	7.83629943768857
N	11.65841468013044	1.85310706836552	1.79671169325888
N	7.64595389407966	2.69081711781826	9.04398959375464
N	10.80571608114849	3.89981281149087	0.12909374591847
C	6.59895514166292	7.47478592938549	3.48686563610151
C	6.13631041360687	7.29494747794340	4.77026468771791
C	6.13929066916108	5.97894260586243	6.90063262828770
C	8.14520288553800	1.64650970857244	9.70480165646811
C	9.09102711720862	0.80003265704966	9.09233048274550
C	7.56892238387218	4.03145178416360	7.01062335543919
C	8.96906731935365	5.80831152561732	1.09025217396706
C	10.37948248523649	3.74106573735334	1.40621133558829
C	8.05012040942013	6.72033850324257	1.59051998413576
C	12.11403795133584	1.95381434369476	0.53773717139444
C	8.10612546106616	2.86575296081187	7.78085796557819
C	9.38942406773158	4.73523927877003	1.92795422880032
C	6.59553788850811	6.19277807774642	5.57215626301203
C	11.68205801192252	2.99546865257040	-0.30752331643030
C	7.55307963085372	6.56452991167157	2.91245676774856
C	6.62177439564573	4.89917165903679	7.62705929478392
C	7.55068787740822	5.25472024799368	5.04399135079414
C	8.04296505714299	5.44565787150840	3.67346249413769
H	9.37502676680220	5.89543563316365	0.07377336306724

H	6.29375920191740	4.70172340444179	8.65613698173134
H	7.70161121267772	7.56373673227571	0.97457880004881
H	5.40712651602949	6.67252422186009	7.34228929008805
H	12.83554394485326	1.19008752523614	0.20645165188565
H	5.40548477611783	7.99383895100708	5.20558933961268
H	6.24497665066272	8.32094668243304	2.87748935379721
H	9.52148368112341	-0.07141716964968	9.61080716429421
H	12.05540233067177	3.08832827550835	-1.34242910504581
H	7.79693089873434	1.46832100278322	10.73714231929414

36

Coordinates from ORCA-job (Cl2BTPhen)_num_water_BP_ORCA4

N	8.93786684463014	4.56971323523529	3.18932473906926
N	8.99145766412181	2.03505190311981	7.11252069461555
N	10.79335662508954	2.75405824978718	2.29198050675425
N	8.00511595772305	4.20489268897459	5.74815239026515
N	9.49369345310840	0.98610420973617	7.76984332782578
N	11.67700918520645	1.84949516098331	1.86122943007974
N	7.67402880452772	2.66532523585880	9.02263819800082
N	10.83804700651305	3.88266203067995	0.16937250740902
C	6.59831734846871	7.47567179643905	3.48131032562634
C	6.12781163397085	7.29195222529116	4.77284369197818
C	6.13260279165101	5.95623877861183	6.90672657375604
C	8.18016552736950	1.61617032564069	9.67093908415539
C	9.10667163268887	0.76449562317321	9.03623295117783
C	7.56682641962037	4.01113663026419	6.99610988096870
C	8.99095926785376	5.79589408014111	1.10136641093206
C	10.40046411966244	3.73138726174173	1.44263810358744
C	8.06862485576190	6.71181740081730	1.58625583129056
C	12.14405400442342	1.94120560699874	0.60583013222649
C	8.10862441854323	2.83748222076014	7.75101200572755
C	9.40411808779987	4.72849624178013	1.94654162413765
C	6.58450471047734	6.18494604783950	5.57957605496037
C	11.71983350791789	2.97593479244913	-0.25196499445490
C	7.55902716504309	6.56536464307459	2.90405667943436
C	6.62057401214820	4.87108120159617	7.62031006974959

C	7.54149002848684	5.25574344458828	5.04143563257454
C	8.03958050550058	5.45049332291512	3.67500081557062
H	9.40350627996608	5.88096532027730	0.08754086055860
H	6.29270138274669	4.66732801949175	8.64805960732118
H	7.72847996673843	7.54957696277810	0.96177462217361
H	5.40092460274562	6.64077049027368	7.35797238826944
H	12.86909691336415	1.17517315780951	0.28791474545152
Cl	4.96847019416167	8.40477068607401	5.44811576597012
Cl	6.03286913115937	8.81997881353915	2.52659526588383
H	9.54496935068338	-0.10906488807482	9.54447357798275
H	12.10333522704631	3.06180267378505	-1.28361227484885
H	7.85259737308027	1.43780240554909	10.70997677381941

36

Coordinates from ORCA-job (F2BTPhen)_num_water_BP_ORCA4

N	8.94729054680013	4.56642734605234	3.17722600753576
N	8.97898272596048	2.04446784286063	7.15679447593430
N	10.79423682080817	2.76282962871595	2.24583464359831
N	8.00548318908458	4.19821475797985	5.76221621315691
N	9.47367447499491	1.00035578905587	7.82728209903077
N	11.67636507779964	1.86403117721224	1.80027337109101
N	7.63982006857935	2.68820701299390	9.04717324106004
N	10.83208874996592	3.91947816206866	0.13828921400499
C	6.61015727854544	7.45666390837990	3.48754105242317
C	6.14008035799045	7.27218281142634	4.77017928392442
C	6.12132252856356	5.96735050954679	6.89840418639823
C	8.13868616544588	1.64381919284541	9.70859908671445
C	9.07201784322771	0.78739090489225	9.09057565220092
C	7.55481860046438	4.01892436366270	7.00897127592427
C	8.98405654985063	5.81706951152631	1.09725173206862
C	10.39860019233836	3.75191562653253	1.41117131241504
C	8.06112557293983	6.72763368755779	1.59162815779169
C	12.13949235842725	1.97232978134926	0.54477295064628
C	8.08821210037497	2.85138207275487	7.77891114166161
C	9.40382723063811	4.74169995642873	1.93198085808262
C	6.59041632339514	6.17543150104116	5.57469297381105

C	11.71232688198108	3.01827069325674	-0.29777474300459
C	7.56504545616127	6.55746256251304	2.91089420865636
C	6.60410328052021	4.88558443444292	7.62099335463102
C	7.54968768563962	5.24387201555047	5.04326524376684
C	8.04915633295918	5.43954067547885	3.67623806440847
H	9.39373764348760	5.90924623123602	0.08289448520997
H	6.27068788809307	4.68688023770121	8.64793153844340
H	7.71354178800549	7.57082009572377	0.97849158542198
H	5.38717702752019	6.65807442778523	7.33625647207829
H	12.86355920168189	1.21062059665535	0.21447375291114
F	5.24347902641995	8.13169122723961	5.28505422364616
F	6.17137028002004	8.49533336008512	2.75558895093703
H	9.50430371912657	-0.08270956708174	9.60977561212730
H	12.09216644984668	3.11764896316600	-1.32958593388786
H	7.79972058234224	1.47330550136393	10.74532425518004

3

Coordinates from ORCA-job (H2O)_num_water_BP_ORCA4

O	15.58411088537149	19.06045545857322	-2.20750491250578
H	16.31628113374366	19.23966578927341	-2.82990249008114
H	14.84349998088484	18.89705775215337	-2.82422359741308

36

Coordinates from ORCA-job (I2BTPhen)_num_water_BP_ORCA4

N	8.95396516379251	4.54814712788926	3.18708651277959
N	9.00645392702413	2.02620249243963	7.14264045951561
N	10.80691523075256	2.73864556960789	2.26875006817091
N	8.02020344791197	4.18334723948931	5.75508815201022
N	9.50180124528932	0.97961557103539	7.80891377503934
N	11.69147369219578	1.83960587157924	1.82834328702073
N	7.66305000478291	2.65867144434899	9.03396952148970
N	10.84418576037550	3.88516377891467	0.15546243368024
C	6.60821178100993	7.46004453583924	3.48854973907341
C	6.14183890579699	7.27777391494656	4.77005737962845
C	6.15067200765655	5.94341885496270	6.88911264744624
C	8.16246066286691	1.61209784452763	9.69128039243035

C	9.09782423752854	0.75977501731654	9.07033573327331
C	7.57679086879552	3.99669558008112	7.00226057537132
C	8.99379535316198	5.78524590380636	1.10812656640328
C	10.41028812092516	3.72239910514006	1.42872022649784
C	8.07194047707821	6.69347581855627	1.60710432854684
C	12.15564187382176	1.94318259831948	0.57277106838881
C	8.11315157378509	2.82785562266308	7.76720564381730
C	9.41484793385888	4.71401413946742	1.94329636122586
C	6.60418823555012	6.16944051959410	5.56492342982981
C	11.72682410651134	2.98401023674878	-0.27548269228888
C	7.56586336214668	6.54499683821515	2.92312308052810
C	6.62964605752515	4.86279872155493	7.61480974900935
C	7.56065163119094	5.23142496872158	5.04172503337856
C	8.05627337552865	5.42520053361434	3.68038368523835
H	9.39874397004135	5.88024194990168	0.09213841995011
H	6.29382548252200	4.66866841425604	8.64183189202755
H	7.71936952426175	7.53699269012563	0.99747478506940
H	5.41771247428992	6.63742758345119	7.32357996687790
H	12.88192930075988	1.18184289682473	0.24657397679260
I	4.77324758813132	8.59042496461107	5.57659322895002
I	5.94557999338155	9.04800089753926	2.35432075354952
H	9.53001369113728	-0.11232890840585	9.58620838212627
H	12.10744938871401	3.07898632196462	-1.30740749497257
H	7.82282154989787	1.43635834035192	10.72689793212452

4

Coordinates from ORCA-job (NO3)_num_water_BP_ORCA4

O	11.37923505351828	4.50595767425207	4.61114299117613
O	10.94283337126837	3.70994884533768	6.60726538981088
O	12.34593845769951	5.37938100591393	6.37501832894225
N	11.55613911751384	4.53161847449631	5.86466429007074

49

Coordinates from ORCA-job Am(Br2BTPhen)(NO3)3_num_water_BP_ORCA4

Am	9.73324905557567	2.59064661390969	4.67148506765334
N	8.90409833580310	4.57487857550395	3.18701658958687

N	8.96454585027337	2.04907779478825	7.06158654864913
N	10.75304713512829	2.76787459361907	2.32015060528011
N	8.01700298529296	4.24241632902004	5.74707645110022
O	7.38434410783200	1.56504977878480	4.73615007325489
O	8.38079141725012	1.54757196185788	2.80463567076883
O	11.86447957185331	1.33092062932688	5.15259054350390
O	10.12709498754207	0.10213946325561	4.68055242509142
O	11.56155676097416	4.36846039816160	4.50413151684040
O	10.96682431265716	3.80712311071834	6.51776084657203
N	9.49055989979869	0.98743547935508	7.66414312302692
N	11.62673698015769	1.84024139999373	1.94352868835194
N	7.69836831737191	2.64203976485415	8.99676262676754
N	10.86736170606207	3.87252622038210	0.20711905065125
O	12.58082413291764	5.27119925692768	6.22942187084503
O	12.04176384634727	-0.85895917995455	5.16762366115074
O	6.39313897697834	0.63372680177043	3.00933626983037
N	11.37025466930940	0.15323343452949	5.00582326531759
N	11.73811278441509	4.51381555214668	5.76695382338561
N	7.34612116011410	1.22473329040676	3.50019833926282
C	6.57800280101927	7.49632331009035	3.47649241658930
C	6.11155812826604	7.31570419040014	4.77081031739603
C	6.13827527983320	5.97517703013636	6.91118462072274
C	8.21584776401804	1.57477055383497	9.60349623515093
C	9.13290223051898	0.73721356584600	8.92941465544817
C	7.57165671213754	4.03282636732370	6.99892559815158
C	8.97619265110170	5.79689184857146	1.10092338788482
C	10.38380732673923	3.74492258295423	1.45956506642747
C	8.05060342650377	6.71334576513592	1.57988444701307
C	12.13244156769178	1.91304777367565	0.70634436311084
C	8.10199916184643	2.85172624801936	7.72709069170354
C	9.38495853594559	4.73311055908722	1.94027710843810
C	6.57697039189119	6.21096786079212	5.58140121574557
C	11.75189255699866	2.95162858841998	-0.17301493990159
C	7.53859951912671	6.58131531545597	2.89763152874628
C	6.62879444788696	4.88695639620579	7.61934253993073
C	7.53330524135769	5.29087593262157	5.03637132867446

C	8.01313266831696	5.47356215042036	3.67503250590293
H	9.39299050372630	5.88154731280370	0.08918542150964
H	6.30153244262606	4.67525511377927	8.64532408801107
H	7.71178039583820	7.54354181737577	0.94522293263332
H	5.40886544889234	6.65467470757628	7.37287723982334
H	12.85028106718706	1.13008669204982	0.41786016899541
Br	4.84982214952545	8.51075979054888	5.52261683396830
Br	5.96950957970254	8.94555662033353	2.42058696777362
H	9.58540874525885	-0.14531329194699	9.40651063905251
H	12.17366270755640	3.02283265940571	-1.18968235136873
H	7.90977555483263	1.36697426972523	10.64251291557564

49

Coordinates from ORCA-job Am(BrHBTPhen)(NO3)3_num_water_BP_ORCA4

Am	9.71753232405926	2.59431343797666	4.66980907410130
N	8.89333347241766	4.58494046831977	3.18443079870471
N	8.95247485250836	2.05812433970558	7.06869360705759
N	10.73397278485205	2.78309986598465	2.31471592961777
N	8.00874100661690	4.23871535002799	5.75272179975989
O	7.36956882652507	1.56526415260431	4.73404208483727
O	8.37230634304714	1.54310439059499	2.80543374173949
O	11.85225583870898	1.33090464564393	5.15472133472891
O	10.10913219668702	0.10246308595303	4.70037742215256
O	11.56014934758346	4.35852272098332	4.49653083418145
O	10.95340007283531	3.81607412301395	6.51197275395935
N	9.48383761135415	1.00002864621716	7.67232049833078
N	11.61052103827111	1.85711891224561	1.94105297490981
N	7.69099762395240	2.65201003774041	9.00714946101575
N	10.86174956037386	3.89561207297376	0.20694576465232
O	12.56417200601397	5.28300194453636	6.21928434506209
O	12.02317067098029	-0.85908244392337	5.18786346631297
O	6.37866040969362	0.64058375058488	3.00337088518808
N	11.35367362655857	0.15364786169059	5.02001742904527
N	11.72731700377934	4.51769283249450	5.75901677771524
N	7.33334812670751	1.22572307188461	3.49787098973048
C	6.56735966540331	7.49389433209459	3.50624685323811

C	6.10696575672308	7.31044889760339	4.79107642209480
C	6.12809391618299	5.98727650345498	6.91310081575474
C	8.21327021778541	1.58733256940953	9.61416902577925
C	9.13149974122448	0.75150905046313	8.93965692616058
C	7.55898445139901	4.04003245172084	7.00584011118133
C	8.96256055733661	5.82002249809777	1.10383381278876
C	10.37139115170356	3.76425645039695	1.45606256061215
C	8.03581177198947	6.73371361457557	1.58880807323793
C	12.12439711592967	1.93457325068515	0.70725621957361
C	8.09005174064753	2.86109780033216	7.73559374290486
C	9.37174420336079	4.75188018081689	1.93824069101422
C	6.57523978007180	6.20936130323318	5.58337951881719
C	11.74815530326942	2.97542286339456	-0.17110392905314
C	7.52663901796406	6.59308086102993	2.90498070020904
C	6.61604398845099	4.89957374193142	7.62526211745837
C	7.52776826763646	5.28758157570713	5.04163016147571
C	8.00207534354054	5.48017313826596	3.67958321146107
H	9.37993195848834	5.90900602078145	0.09263408974588
H	6.29199499168225	4.68735494165769	8.65233866554973
H	7.69318710868495	7.56858476234549	0.96126845595681
H	5.39893795354385	6.67434235998405	7.36690121617996
H	12.84533155263351	1.15367979640502	0.42087686414560
H	5.37557863394947	8.00281744698256	5.23165799188063
Br	5.91700591008436	8.97200734242982	2.50381597412278
H	9.58887237181084	-0.12790202599423	9.41784493563397
H	12.17496102634880	3.04906413929635	-1.18550142353528
H	7.91067775862845	1.38038386564598	10.65443922280835

49

Coordinates from ORCA-job Am(BTPhen)(NO3)3_V2_num_water_BP_ORCA4

Am	9.73078628011924	2.58114328318420	4.66920776448382
N	8.90574972810328	4.55924380070767	3.17839742788556
N	8.94274059622903	2.04050332964059	7.06701606277389
N	10.76251755260889	2.78177837643165	2.31492738024684
N	8.02776031770996	4.23397544298517	5.75720225235404
O	7.36230776147674	1.59569922683494	4.68402198402908

O	8.42436613809379	1.49933336440008	2.78923758154821
O	11.87494079108585	1.32396649994600	5.11779604948110
O	10.11972991107336	0.09030100813720	4.72852888006604
O	11.53427147180649	4.39216041675063	4.53395761726271
O	10.98183208962246	3.75490254580943	6.53790050104438
N	9.46228984168318	0.97587807389275	7.67026181460037
N	11.64482389350999	1.86091983382151	1.94167584268603
N	7.70424698757738	2.65717927173021	9.01418199231209
N	10.85927795450053	3.87221430953017	0.19267273334833
O	12.56790632034665	5.25308541369423	6.27198394111694
O	12.04624486989273	-0.86572167859959	5.17658691490224
O	6.40818404479571	0.64046872676105	2.94929176045435
N	11.37265675550834	0.14487423898073	5.01310568319391
N	11.72887675198827	4.49764462789819	5.79784906390660
N	7.35887765938098	1.22173686710463	3.45724479574661
C	6.57948432101679	7.47516152867941	3.48185231615619
C	6.12868587046463	7.30455184131065	4.77087765576774
C	6.15825836697951	5.99689991205609	6.91181451458828
C	8.21378672358968	1.58530489965832	9.61949580895964
C	9.11461501500882	0.73493383888948	8.94036516335714
C	7.57813234630779	4.04256341111984	7.01083584694874
C	8.95452550520761	5.79579372505700	1.09220045220432
C	10.38022312676149	3.74951872645202	1.44745975341652
C	8.02611884152022	6.70655578373636	1.58143495297921
C	12.14704925835041	1.93038901085116	0.70259945733361
C	8.09791361068195	2.85810784847063	7.73973568041305
C	9.37474313208238	4.73031678707263	1.92852800274582
C	6.59759128910119	6.21053423120310	5.57874599782332
C	11.75275338833434	2.95795981134905	-0.18294950428817
C	7.53184912807982	6.56424945304249	2.90482760170416
C	6.64240081718517	4.91020753011909	7.62945051322613
C	7.54687990738258	5.28033569762779	5.03988119396209
C	8.01740045932385	5.45675214178284	3.67509403533942
H	9.36726507959335	5.88058602136243	0.07859539538269
H	6.31958962103298	4.70425531311684	8.65817490216413
H	7.67122629514539	7.53894501361048	0.95533204931276

H	5.43339989113915	6.68921958306540	7.36512545909501
H	12.87267467205990	1.15323044412711	0.41783574047480
H	5.39847640790940	8.00190595548496	5.20813649398863
H	6.21560705942337	8.31228300107084	2.86767598130691
H	9.56210352252629	-0.15039291100103	9.41702880993890
H	12.17046970307396	3.02572782947885	-1.20159837952166
H	7.91455492360510	1.38442359156473	10.66194806177748

49

Coordinates from ORCA-job Am(Cl2BTPhen)(NO3)3_num_water_BP_ORCA4

Am	9.72869671329167	2.59242293768973	4.66967742777298
N	8.90226705323408	4.57581569937684	3.18434285234560
N	8.95894246101341	2.05428526371825	7.06382955305957
N	10.74991388833550	2.77385425212028	2.31734676752906
N	8.01317808893790	4.24277806196853	5.74846971438636
O	7.38025985937707	1.56656894836908	4.73490149536227
O	8.37602099505006	1.55252594720720	2.80300634967458
O	11.86131807222005	1.33263442925997	5.14878129783566
O	10.12352582593815	0.10370978653822	4.67775186502718
O	11.55586365187183	4.37201066830698	4.50359202050204
O	10.96195207159518	3.80826825227444	6.51676886568055
N	9.48567545770802	0.99321321803897	7.66668514892114
N	11.62582586180791	1.84769148875347	1.94213701369094
N	7.69415885609013	2.64828526324590	8.99975905768313
N	10.86457983986354	3.87806828528591	0.20409712713708
O	12.57497590654015	5.27362389863011	6.22958806559420
O	12.03907327074434	-0.85718747767091	5.16157671524550
O	6.38878506888568	0.63765869796568	3.00690202687772
N	11.36700731034390	0.15490095724151	5.00146117322921
N	11.73259484518943	4.51622587591184	5.76651804735213
N	7.34173611782588	1.22823657597385	3.49836927081397
C	6.58062182246429	7.49436930983070	3.47679914465504
C	6.11241658983376	7.31275205380215	4.77072034264067
C	6.13512650598171	5.98365256515090	6.91034348188974
C	8.21234442810315	1.58151201642321	9.60675105584673
C	9.12912312732454	0.74374027720541	8.93256005178642

C	7.56653581017333	4.03799315290427	7.00081858983469
C	8.97083920082341	5.80351889127143	1.09934611455608
C	10.38052375188927	3.75044813680525	1.45624437070158
C	8.04504107540705	6.71951069577899	1.58007288677966
C	12.13287526410998	1.92119769441012	0.70543537810474
C	8.09670740698331	2.85721644370717	7.72965454181312
C	9.38099271142616	4.73807560603178	1.93718165098008
C	6.57691045650153	6.21046294605304	5.58094167081622
C	11.75080030024014	2.95834121474484	-0.17490702270096
C	7.53901162925373	6.58058315314234	2.89849942921718
C	6.62535123049161	4.89591170129696	7.62043217229731
C	7.53201054619516	5.29055543071391	5.03609907375586
C	8.01214410387673	5.47309642281318	3.67496354675338
H	9.38684717528760	5.88857404078599	0.08728060139609
H	6.29895869958666	4.68591343378582	8.64707627391010
H	7.70076323168247	7.55112447682843	0.95031884409785
H	5.40810210820268	6.66827025120580	7.36822612518382
H	12.85315993234545	1.14006091120630	0.41810432044242
Cl	4.95141800421647	8.41435366015216	5.44251164775537
Cl	6.01724125822239	8.83003948565115	2.52218545727457
H	9.58227554783595	-0.13815845259869	9.41020530681247
H	12.17296267231718	3.02940902306141	-1.19142258448683
H	7.90739019336040	1.37431742763018	10.64623067216581

49

Coordinates from ORCA-job Am(F2BTPhen)(NO3)3_num_water_BP_ORCA4

Am	9.72283322831809	2.60159562825173	4.66877380830881
N	8.90129386416146	4.58249582323986	3.17741781089131
N	8.94550589376322	2.06834079990025	7.06990545073104
N	10.74221627463187	2.79114705047488	2.30796847732322
N	8.00838231481705	4.24782549004640	5.75428480165613
O	7.37375691990341	1.57696052900673	4.73333905106128
O	8.37038639212936	1.56228090493440	2.80197086563071
O	11.85498017570403	1.34151627108368	5.14693791249343
O	10.11651524710977	0.11292202970688	4.67770123776568
O	11.54893658675226	4.38198111123852	4.50331939050208

O	10.95599495251040	3.81637701712691	6.51622334807030
N	9.47074268786271	1.00721773323198	7.67353273433905
N	11.61883767922592	1.86610570738812	1.93245333330437
N	7.67894503089690	2.66284609214177	9.00563296407973
N	10.85691072465916	3.89603779320802	0.19409354559570
O	12.56909660062157	5.28176421877873	6.22967032946280
O	12.03208574905585	-0.84838967705461	5.16081019573246
O	6.38308196448520	0.64749549495816	3.00514602910170
N	11.36026930413972	0.16375864265802	5.00063041574247
N	11.72637678957161	4.52488607731224	5.76627710696018
N	7.33583173107979	1.23819782519041	3.49692783799192
C	6.58464984981157	7.48390792470676	3.48085584241727
C	6.12372162007901	7.30734086488826	4.76988622537490
C	6.12518057296154	6.00374497795832	6.90641595351269
C	8.19614903800275	1.59553128708691	9.61274000986488
C	9.11304953433525	0.75758827416046	8.93925037035863
C	7.55506801916436	4.05230256543285	7.00558841804464
C	8.95412169497831	5.82164453202747	1.09408272285197
C	10.37246813271406	3.76804847099988	1.44601724815961
C	8.02702130753603	6.73544480289865	1.57867194372573
C	12.12646972403327	1.93997323585227	0.69579375371233
C	8.08267189350432	2.87186581287211	7.73593762806313
C	9.37205954194227	4.75349092150652	1.92850297317971
C	6.57962726350212	6.21513608905875	5.57921484277164
C	11.74397207429386	2.97677026106050	-0.18453034059886
C	7.53508936148821	6.58107861248346	2.90032216662823
C	6.61164175297720	4.91542184007269	7.61946690348966
C	7.53324315173238	5.29282026678308	5.03612751790452
C	8.01169223102756	5.47422629644113	3.67515582053552
H	9.36769244246358	5.90828822901677	0.08104288685269
H	6.28332951045534	4.70738010426604	8.64597356798753
H	7.67498408101084	7.56871992032136	0.95514407214461
H	5.39476632641888	6.69020110031223	7.35598602241038
H	12.84739188239824	1.15931225663862	0.40877182985138
F	5.23390261516945	8.16551744877799	5.28393911187816
F	6.13971487028747	8.51157932626526	2.74673911622875

H	9.56535427765691	-0.12465419771385	9.41705048359735
H	12.16666635578939	3.04821041765033	-1.20082632033305
H	7.89016676286648	1.38817879535212	10.65189758264167

49

Coordinates from ORCA-job Am(I2BTPhen)(NO3)3_V2_num_water_BP_ORCA4

Am	9.73426881761684	2.58140018333376	4.67043099238060
N	8.91121727948811	4.56134240788222	3.18396546704934
N	8.96503841021765	2.04382228654969	7.07091373543750
N	10.75584907863225	2.76392636245757	2.31275077794363
N	8.02372321262412	4.22867211862436	5.75074341601934
O	7.39060900784494	1.54950137205981	4.74300450362836
O	8.38107096428281	1.53351783760619	2.80850078187206
O	11.87114573035410	1.32812481995990	5.14955557876366
O	10.13667622571508	0.09362852788437	4.67983382669318
O	11.56371082999128	4.36191378256000	4.49792546932774
O	10.97076923003149	3.80167029076810	6.51251444043762
N	9.48852147688888	0.98294476210646	7.67675078528748
N	11.63505327221090	1.84133410439907	1.93661992677186
N	7.69393016773480	2.64090309860669	9.00274077658294
N	10.86465834726325	3.86946031681346	0.19869769096134
O	12.58564334002803	5.26373186767726	6.22259946160575
O	12.05643077280628	-0.86121363717834	5.15980917943993
O	6.39996385316165	0.60654996965001	3.02213940451786
N	11.38043701968992	0.14876628170146	5.00238969372878
N	11.74163518050292	4.50734389589442	5.76042790956291
N	7.35055856198480	1.20567888510903	3.50785790992751
C	6.58708142609809	7.48902913823698	3.48404149073558
C	6.12475337098927	7.30977745786375	4.76911974756902
C	6.14840545092493	5.97373602948369	6.89141897249286
C	8.20784039553689	1.57314669069981	9.61177897241003
C	9.12584313461553	0.73397215801716	8.94114757111933
C	7.57372642134653	4.02736350283150	7.00213386271947
C	8.97160003241297	5.79701108774389	1.10756791940297
C	10.38358004094942	3.74029434066291	1.45193152402174
C	8.04800133328517	6.70877388265607	1.60099804788778

C	12.14134309606536	1.91746598905323	0.69952353210341
C	8.10086965550042	2.84797654331115	7.73372006665382
C	9.38593845963422	4.72689678657533	1.93620601948125
C	6.59313689607227	6.20374324114192	5.56603393980432
C	11.75340000680190	2.95225614184834	-0.18101913310467
C	7.54131458314761	6.56949585033587	2.91673296809782
C	6.62911750726140	4.88799909182993	7.61110025719190
C	7.54533881726190	5.27474130502529	5.03492642072310
C	8.02357218561976	5.45731314784242	3.67935588910031
H	9.38420232217809	5.88897710242984	0.09463812227860
H	6.29484285075839	4.68563846473117	8.63679595152350
H	7.70153098368735	7.54951523612630	0.98481104859630
H	5.41529152282186	6.66210316974768	7.33344692642144
H	12.86595599656872	1.14052464306814	0.41210803059219
I	4.76394475144521	8.61491991223748	5.57655108975049
I	5.92564104157058	9.06532224230317	2.35024034869468
H	9.57519475969143	-0.14878062776868	9.42081623361050
H	12.17406574370419	3.02397538325410	-1.19813919486988
H	7.89840243498041	1.36622555424608	10.65007664705282

49

Coordinates from ORCA-job Eu(Br2BTPhen)(NO3)3_num_water_BP_ORCA4

Eu	9.72841889016871	2.58161717022575	4.66928353976632
N	8.90189919571141	4.56524204347367	3.18412956710398
N	8.94834746736590	2.02564594620686	7.06052643179242
N	10.77341292280583	2.76874508757990	2.32335012368392
N	8.01640233160657	4.23514393913932	5.74822745436961
O	7.34751810138402	1.62135002262435	4.67724878759491
O	8.40362146668785	1.51639916617431	2.77949733873721
O	11.86069194501862	1.34672981284040	5.10195559310268
O	10.10743027087375	0.11171386693867	4.72130830991294
O	11.51122203674969	4.41542427786315	4.54149692597548
O	10.96898986418974	3.77119829192155	6.54645165249327
N	9.47678668208309	0.97224552412299	7.66954235408803
N	11.64891275664970	1.84740127566116	1.94444229089827
N	7.71928643794880	2.65217060373386	9.00744942405197

N	10.85920480478365	3.85740335529173	0.20231069263225
O	12.54461463712672	5.27563336736607	6.27722793975566
O	12.03710617072924	-0.84112848676680	5.15409258708681
O	6.38465808302221	0.67294142498720	2.94626023522898
N	11.36014057660888	0.16860792002021	4.99761672535377
N	11.70618009867439	4.51569374268863	5.80307016604110
N	7.34247026144133	1.24900357686643	3.45262543373820
C	6.58070340201970	7.48874029535408	3.47640551161195
C	6.11713286063676	7.31066689673571	4.77207988301224
C	6.14766974559004	5.97337116551283	6.91457990240794
C	8.23640489702661	1.58815538891959	9.61997220019739
C	9.13933591793668	0.73748421267632	8.94382790743521
C	7.57581481308154	4.02740569397208	7.00031449634501
C	8.97539536005560	5.78642158692960	1.09933024847845
C	10.38730402740981	3.73599672153693	1.45953955928981
C	8.05052200289285	6.70266121433546	1.57881954370790
C	12.14548066882260	1.91300717775966	0.70260549327454
C	8.10495675266682	2.84515137981396	7.72972060322634
C	9.38403821534206	4.72224666772891	1.93964992530394
C	6.58260864866864	6.20616972548819	5.58283500477614
C	11.74855057108305	2.94207970965949	-0.18014576031213
C	7.53876855034121	6.57159553470451	2.89695451244828
C	6.63711655469577	4.88569702910933	7.62337435088498
C	7.53525638519313	5.28228350143885	5.03769424814296
C	8.01346248151865	5.46288138361571	3.67303989218492
H	9.39173834217676	5.87199315131983	0.08751551442717
H	6.31219414802770	4.67737772129330	8.65075739708567
H	7.71290450612761	7.53365783795304	0.94447689799922
H	5.42114638789259	6.65519943601636	7.37750324407142
H	12.86709211852846	1.13368454791322	0.41399608267714
Br	4.86008991314943	8.50886458720260	5.52570329408305
Br	5.97266255881437	8.93735444583609	2.42017104384104
H	9.59585138215187	-0.14052259848319	9.42523828810474
H	12.16053009779102	3.00974311967238	-1.20100260317937
H	7.94279968872857	1.39388353702577	10.66516474506630

49

Coordinates from ORCA-job Eu(BrHBTPhen)(NO3)3_num_water_BP_ORCA4

Eu	9.71938293598496	2.59145544855477	4.67294437664224
N	8.89494523027137	4.57705732390642	3.18297191579029
N	8.93803167246973	2.03640848500715	7.07101519072771
N	10.76114797013791	2.78701669709360	2.31945593253760
N	8.01405169069974	4.23542806634830	5.75634730364960
O	7.34024949687094	1.62225433952223	4.67999748384734
O	8.39846189696565	1.52194611210848	2.78306166516880
O	11.85229120869881	1.35370667557340	5.09994657393476
O	10.09479965168592	0.12022236639979	4.73358676667595
O	11.50292976282839	4.42481740021245	4.54716293168028
O	10.96895762959280	3.77282987776361	6.55168374750217
N	9.46687227182469	0.98417790177197	7.68109148937553
N	11.63517336664226	1.86473660976266	1.93930063089657
N	7.70641110924349	2.66178176098449	9.01747043794675
N	10.84850019150917	3.87743072333073	0.19901906940162
O	12.54260644541609	5.27917311363786	6.28225603435064
O	12.02475516950534	-0.83416173579976	5.16144503305817
O	6.38194830050564	0.67229005880407	2.94729884813822
N	11.34882231030212	0.17610980689604	5.00349242490364
N	11.70266732479695	4.52034978216649	5.80855854620440
N	7.33755038842010	1.25090876509569	3.45527265053593
C	6.56988424660825	7.48549956545536	3.50474428951036
C	6.11197339273538	7.30471129451778	4.79077020221103
C	6.13633510051647	5.98529237289818	6.91496733723289
C	8.22426892725391	1.59831716787832	9.63025140558394
C	9.12912284040651	0.74888316907209	8.95531191992766
C	7.56550421075886	4.03680476283840	7.00821069238326
C	8.96098283963321	5.80735480156398	1.10120105636326
C	10.37620897454393	3.75525622729330	1.45585359435153
C	8.03457808306852	6.72082982730248	1.58626998908376
C	12.13179065338774	1.93065492444585	0.69741612047814
C	8.09280421602921	2.85612763523291	7.73982012538166
C	9.37268296552055	4.74091186200363	1.93817897822707
C	6.58204233681861	6.20539627545109	5.58432815258743

C	11.73696103470777	2.96138251355196	-0.18415683296006
C	7.52807289808155	6.58336990835549	2.90380314104254
C	6.62361330815924	4.89852608951866	7.62832426689119
C	7.53388872831919	5.28164559245301	5.04415353252413
C	8.00603343500359	5.47138307985060	3.67869652982280
H	9.37610243218103	5.89596644084179	0.08911191083652
H	6.29875247727225	4.68837021667390	8.65551859916487
H	7.69025514169677	7.55406578608816	0.95738146123148
H	5.40727800818517	6.67279825301735	7.36844366786277
H	12.85197547105989	1.15020671084570	0.40816901348386
H	5.38055200852439	7.99724705682530	5.23094195974329
Br	5.91712656945213	8.96049018383819	2.49987222947925
H	9.58659766280636	-0.12849276922245	9.43696462992115
H	12.14943857117162	3.02989943652498	-1.20477741138672
H	7.92946344172589	1.40359503574355	10.67508538605266

49

Coordinates from ORCA-job Eu(BTPhen)(NO3)3_num_water_BP_ORCA4

Eu	9.72994666413055	2.58263940663582	4.66918578133846
N	8.91275120141274	4.55807075702655	3.17924672045670
N	8.94308709061945	2.02786502127431	7.07108144817845
N	10.77498675907095	2.77699493808857	2.31303126983469
N	8.02733659133827	4.22772497559488	5.75712050160495
O	7.34952065437327	1.61840892519893	4.67037604232158
O	8.41561746510367	1.50294312412945	2.77902469223057
O	11.86285508861949	1.34113605132048	5.09675492829250
O	10.10318205456537	0.10937805261767	4.73811894673679
O	11.52267293106062	4.40927465089325	4.54914631313452
O	10.97936689528470	3.75968785838583	6.55170589044741
N	9.46685484086279	0.97246100298475	7.68028258945113
N	11.65080647538496	1.85633920433342	1.93426071854780
N	7.71140336810081	2.65516315591710	9.01720687850947
N	10.86057330393351	3.86487849859920	0.19071077305796
O	12.55700734689304	5.26311320315291	6.28792994703005
O	12.03194123420210	-0.84685489915891	5.16748316961993
O	6.39360125407256	0.66492997318430	2.93802745062148

N	11.35752338509291	0.16415079314906	5.00611639426439
N	11.71745335367245	4.50520658020432	5.81093200151562
N	7.35047077796058	1.24090681212149	3.44750217024005
C	6.58359598206860	7.47019394613868	3.48212821695394
C	6.12969930102019	7.29736746979180	4.76972563183839
C	6.15534887577416	5.98588737380063	6.90917914294812
C	8.22346732911590	1.58798869846507	9.62857073906841
C	9.12564132509950	0.73603141780291	8.95342420047571
C	7.57792005148622	4.03327949723212	7.00867801867542
C	8.96332600154800	5.79340503225362	1.09430747316642
C	10.38752299412957	3.74432654289772	1.44800629195302
C	8.03488765197462	6.70381861724086	1.58359304043940
C	12.14855181815329	1.92170363406438	0.69273337423580
C	8.10035482402432	2.85000499458618	7.74032866345283
C	9.38278879158261	4.72705543772507	1.93121920410458
C	6.59732589974272	6.20264502369480	5.57742400920505
C	11.75128979794940	2.95021099508308	-0.19027469771319
C	7.53793314922785	6.56083111809211	2.90590252939241
C	6.63951164549852	4.89961777767349	7.62703076178280
C	7.54902312740546	5.27343669122083	5.04067268784092
C	8.02364151425154	5.45244458315730	3.67522576647417
H	9.37592466549490	5.87984515376266	0.08081546546869
H	6.31398226193578	4.69344327604210	8.65483205222908
H	7.68137974633409	7.53678407908485	0.95738534798760
H	5.42868742978858	6.67655542300280	7.36224061009341
H	12.87068871389324	1.14254554278495	0.40495211420181
H	5.39826581533785	7.99389190647363	5.20627336829369
H	6.21989612590269	8.30746591619465	2.86806308703238
H	9.57833328746659	-0.14433415902832	9.43415060148470
H	12.16428541551650	3.01824764769755	-1.21078830168065
H	7.92593372252249	1.39249527741110	10.67250797316059

49

Coordinates from ORCA-job Eu(Cl2BTPhen)(NO3)3_num_water_BP_ORCA4

Eu	9.72790438479775	2.58425018253865	4.66921210098885
N	8.90224871737120	4.56742655821771	3.18226021413282

N	8.94414253364288	2.03204847832653	7.06347220717752
N	10.77036070515702	2.77534426307108	2.31937447858333
N	8.01615364540132	4.23752990979727	5.75082711721354
O	7.34642065917157	1.62318161174087	4.67772247838852
O	8.40177569913136	1.52059533203667	2.77939728936800
O	11.85968434425556	1.34883095343592	5.10192438487144
O	10.10619016255876	0.11464701484215	4.72128611022793
O	11.51135565450143	4.41847705453739	4.54154581635147
O	10.96760903039065	3.77462714449714	6.54604283127008
N	9.47138842569958	0.97799458601647	7.67227869273339
N	11.64566978833955	1.85407211053707	1.94013902637307
N	7.71215352395242	2.65661319948794	9.00951440421032
N	10.85611892159428	3.86421770480032	0.19814834159972
O	12.54076245116089	5.28182261322851	6.27822503653316
O	12.03545544996173	-0.83912255296664	5.15411814052911
O	6.38203805204050	0.67905681295304	2.94526258329542
N	11.35893659379877	0.17083190195436	4.99762857246559
N	11.70431777191810	4.52007049488007	5.80332546935160
N	7.34062034567629	1.25324720836143	3.45244920917947
C	6.58366797651724	7.48746476284245	3.47676003098193
C	6.11781873642707	7.30792946730324	4.77217237005832
C	6.14515543831904	5.98210720507817	6.91356340301786
C	8.22793002853634	1.59157118603474	9.62144780636700
C	9.13204528455490	0.74167724002619	8.94575120270317
C	7.57270894088751	4.03376860030414	7.00268764247874
C	8.96813498901601	5.79206963464357	1.09691577611585
C	10.38406325541825	3.74256493520109	1.45531469349152
C	8.04317570767502	6.70784496864635	1.57847652285704
C	12.14260741687344	1.91990089964384	0.69847706165404
C	8.09993769817206	2.85111799527218	7.73263301667702
C	9.38053005723900	4.72775017419287	1.93656051653709
C	6.58384638816845	6.20628919172853	5.58252325606535
C	11.74587035082739	2.94927225092560	-0.18414318336230
C	7.53983743221096	6.57133302687096	2.89827711840082
C	6.63448498574507	4.89493995839215	7.62429145800827
C	7.53654176376572	5.28343962699389	5.03829356972604

C	8.01450387943778	5.46368166307918	3.67376189432790
H	9.38203140875233	5.87743161331261	0.08405528070059
H	6.30924649055194	4.68792201072449	8.65186896985464
H	7.69770811527617	7.53867562522518	0.94821421736808
H	5.41963301393528	6.66807063300488	7.37203255085967
H	12.86411238290251	1.14043968083565	0.40995986170632
Cl	4.96186815735161	8.41261145359521	5.44578514773111
Cl	6.01783150136288	8.82022246662350	2.52078892038329
H	9.58772001747756	-0.13708758603973	9.42658310023808
H	12.15827177170759	3.01759049471662	-1.20481047115219
H	7.93225595036919	1.39607123852851	10.66583876136042

49

Coordinates from ORCA-job Eu(F2BTPhen)(NO3)3_num_water_BP_ORCA4

Eu	9.72481122937474	2.58997527759752	4.66864981951129
N	8.90112233902986	4.57386074454065	3.17530387253772
N	8.93111366524568	2.04862505019303	7.07048170488249
N	10.76110577824132	2.79277313885665	2.30877881389918
N	8.01206272573461	4.24330671451826	5.75724869728165
O	7.34258958452782	1.63375930081189	4.67816884361267
O	8.39614848280209	1.53008661951548	2.77913877076978
O	11.85648239890784	1.35266951036678	5.09834121713335
O	10.10053928604114	0.11965057572804	4.72434137265638
O	11.50571116916369	4.42527587222091	4.53908740569535
O	10.96261567725218	3.78168989815632	6.54368216574252
N	9.45602845223731	0.99353690470666	7.67890718516455
N	11.63775977224980	1.87312794872071	1.92973855112014
N	7.69535648276925	2.67118712057431	9.01565222709529
N	10.84973600695107	3.88440051552247	0.18831822710028
O	12.53313537486324	5.29176142938466	6.27552956630198
O	12.02977300117713	-0.83540917378577	5.15454251066854
O	6.37488154419169	0.69214027252948	2.94606378274927
N	11.35408347583435	0.17495533277585	4.99758029219011
N	11.69833768744507	4.52815760216529	5.80081496270810
N	7.33492031667727	1.26427376196910	3.45270633297499
C	6.58659009725244	7.47619210508520	3.48063820133641

C	6.12965671501293	7.30299361617535	4.77144373743367
C	6.13594346441188	6.00364637655597	6.91020278257771
C	8.20975461020171	1.60488992750068	9.62696054888478
C	9.11432833971866	0.75541363446278	8.95167834225760
C	7.56164885682792	4.04968062288193	7.00806973122840
C	8.94946572531311	5.80958844296242	1.09077178003741
C	10.37540688197463	3.76094436934359	1.44433638223585
C	8.02314648478852	6.72285735317731	1.57634599412731
C	12.13713886813532	1.94058893855580	0.68890073316927
C	8.08556253286506	2.86726243285925	7.73987844393978
C	9.37029268672253	4.74307319170193	1.92749208296323
C	6.58706120931928	6.21186291909000	5.58120288905584
C	11.74123642066392	2.97053752590691	-0.19315491457529
C	7.53463960252140	6.57122426004238	2.89966298657728
C	6.62167619243661	4.91643358871835	7.62435925642588
C	7.53809676890242	5.28621589903927	5.03872636821932
C	8.01368513159638	5.46466113841599	3.67383242481073
H	9.36061509453936	5.89587601442733	0.07678560301947
H	6.29411094885438	4.71068542546354	8.65152352335636
H	7.66887549695436	7.55507242549457	0.95254108741876
H	5.40878515254223	6.69314947702495	7.36053902349188
H	12.85986012814329	1.16203696963271	0.40100487731429
F	5.24140824859750	8.16142278011685	5.28634140396223
F	6.14115492156039	8.50217697167462	2.74544997296746
H	9.56842972301757	-0.12448445657676	9.43189790226987
H	12.15575813238833	3.04047988267718	-1.21286868350036
H	7.91220311402071	1.40814675052163	10.67059619719925

49

Coordinates from ORCA-job Eu(I2BTPhen)(NO3)3_num_water_BP_ORCA4

Eu	9.73333100744674	2.57640038142776	4.66989620895775
N	8.91288374122533	4.55285661116498	3.18244278671926
N	8.94968816958797	2.02159569782063	7.06849674438807
N	10.77816761829676	2.76627604300544	2.31649940611143
N	8.02618595476676	4.22200126209575	5.75283185788198
O	7.35273858388763	1.61259909634594	4.67870639440080

O	8.40826489393086	1.51002614845939	2.78040588764894
O	11.86578905510237	1.34073309574081	5.10295545301283
O	10.11265706970583	0.10586871235610	4.72311022161120
O	11.51781610450374	4.40937873477264	4.54202980927530
O	10.97440463027144	3.76560698429892	6.54677412408350
N	9.47597702965887	0.96814673730804	7.67903175250970
N	11.65382529721123	1.84590269121330	1.93618836105677
N	7.71561458908131	2.64899576535799	9.01332060373728
N	10.86223977872008	3.85703989851995	0.19527768581791
O	12.54652799598598	5.27379868566109	6.27856370802591
O	12.04218490821961	-0.84712928697531	5.15637757791120
O	6.38847748754927	0.66860502304185	2.94613656812931
N	11.36531888913719	0.16262072767406	4.99932547318994
N	11.71056290416264	4.51128780791559	5.80373900128162
N	7.34712310846840	1.24277265866523	3.45344640799620
C	6.58782103350701	7.47732786186930	3.48309975596761
C	6.12998018518864	7.30187594553229	4.77016868081986
C	6.15965232184018	5.97185689046470	6.89569597054513
C	8.23110821615274	1.58461954443123	9.62680380234188
C	9.13552651287033	0.73371493568285	8.95270709700737
C	7.57996088866275	4.02209730757751	7.00401038197428
C	8.96997964686129	5.78452768301249	1.10534368581713
C	10.39132315532369	3.73397093505843	1.45273782057406
C	8.04442488684334	6.69406144526561	1.59780691439248
C	12.15008120392828	1.91294106713039	0.69419191048188
C	8.10490955956236	2.84135569246719	7.73656857090979
C	9.38812744313273	4.71688701756819	1.93643785044183
C	6.60035292522757	6.19810740904224	5.56800124446230
C	11.75224681910760	2.94249795925761	-0.18775026149192
C	7.54045417743993	6.55700508129519	2.91515887170290
C	6.64076439168033	4.88760439148738	7.61611734345708
C	7.54991354383696	5.26636604141513	5.03720774421799
C	8.02582036094121	5.44636457515251	3.67780215452504
H	9.37820892064361	5.87960040428148	0.09101370044093
H	6.30734818220340	4.68812211267998	8.64256057560275
H	7.69431878300083	7.53121356865376	0.97834564275534

H	5.42980405893122	6.66286835891204	7.33953353987368
H	12.87198915415461	1.13417446204657	0.40478883421793
I	4.77165931817678	8.60497969283692	5.58031838517831
I	5.91976388838090	9.04589491597402	2.34622935399595
H	9.59058634987786	-0.14449442607228	9.43514150459037
H	12.16409347940402	3.01119186028908	-1.20862083665044
H	7.93484777619982	1.39031679081807	10.67125872810166

Table S5 Calculation results of binding energy (hartree)

	E_{BE}	E_{comp}	E_{metal}	$E_{ligands}$	E_{BSSE}	E'_{metal}	$E'_{ligands}$
$Eu(H_2BTPhen)(NO_3)_3$	-0.892	-13049.52	-11077.11	-1971.88	-0.351	-11076.72	-1971.91
$Am(H_2BTPhen)(NO_3)_3$	-0.800	-34054.53	-32082.21	-1971.87	-0.352	-32081.83	-1971.90
$Eu(BrHBTPhen)(NO_3)_3$	-0.886	-15668.90	-11077.11	-4591.25	-0.348	-11076.73	-4591.29
$Am(BrHBTPhen)(NO_3)_3$	-0.867	-36673.98	-32082.21	-4591.25	-0.353	-32081.83	-4591.28
$Eu(F_2BTPhen)(NO_3)_3$	-0.885	-13248.23	-11077.11	-2170.59	-0.350	-11076.73	-2170.62
$Am(F_2BTPhen)(NO_3)_3$	-1.028	-34253.31	-32082.21	-2170.42	-0.358	-32081.83	-2170.45
$Eu(Cl_2BTPhen)(NO_3)_3$	-0.883	-13972.86	-11077.11	-2895.22	-0.345	-11076.73	-2895.25
$Am(Cl_2BTPhen)(NO_3)_3$	-0.882	-34977.94	-32082.21	-2895.20	-0.357	-32081.83	-2895.23
$Eu(Br_2BTPhen)(NO_3)_3$	-0.883	-18288.27	-11077.11	-7210.62	-0.348	-11076.73	-7210.66
$Am(Br_2BTPhen)(NO_3)_3$	-0.850	-39293.33	-32082.21	-7210.62	-0.357	-32081.83	-7210.65
$Eu(I_2BTPhen)(NO_3)_3$	-0.887	-27409.50	-11077.11	-16331.85	-0.349	-11076.72	-16331.89
$Am(I_2BTPhen)(NO_3)_3$	-0.869	-48414.58	-32082.21	-16331.85	-0.357	-32081.83	-16331.88

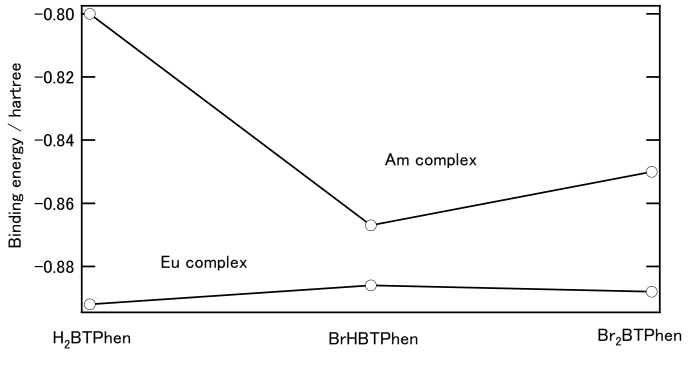

Figure S1 Binding energy for $[M(X_1X_2BTPhen)(NO_3)_3]$.

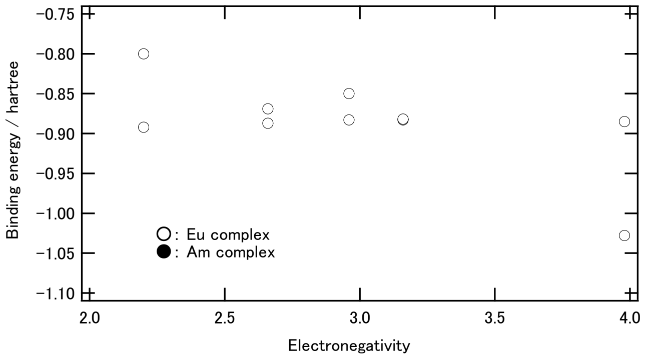

Figure S2 Halogen effect on binding energy for [M(X_2BTPhen)(NO_3)$_3$] (X = H, F, Cl, Br, and I).
